# Thermally reconfigurable metalens

**DOI:** 10.1515/nanoph-2022-0147

**Published:** 2022-05-30

**Authors:** Anna Archetti, Ren-Jie Lin, Nathanaël Restori, Fatemeh Kiani, Ted V. Tsoulos, Giulia Tagliabue

**Affiliations:** Laboratory of Nanoscience for Energy Technologies (LNET), STI, École Polytechnique Fédérale de Lausanne (EPFL), Lausanne 1015, Switzerland

**Keywords:** dielectric nanoresonators, metasurfaces, thermo-optical effects, tunable metalenses

## Abstract

Reconfigurable metalenses are compact optical components composed by arrays of meta-atoms that offer unique opportunities for advanced optical systems, from microscopy to augmented reality platforms. Although poorly explored in the context of reconfigurable metalenses, thermo-optical effects in resonant silicon nanoresonators have recently emerged as a viable strategy to realize tunable meta-atoms. In this work, we report the proof-of-concept design of an ultrathin (300 nm thick) and thermo-optically reconfigurable silicon metalens operating at a fixed, visible wavelength (632 nm). Importantly, we demonstrate continuous, linear modulation of the focal-length up to 21% (from 165 μm at 20 °C to 135 μm at 260 °C). Operating under right-circularly polarized light, our metalens exhibits an average conversion efficiency of 26%, close to mechanically modulated devices, and has a diffraction-limited performance. Overall, we envision that, combined with machine-learning algorithms for further optimization of the meta-atoms, thermally reconfigurable metalenses with improved performance will be possible. Also, the generality of this approach could offer inspiration for the realization of active metasurfaces with other emerging materials within field of thermo-nanophotonics.

## 1 Introduction

Optical metasurfaces are two-dimensional (2D) arrangements of meta-atoms with sub-wavelength spacing that are engineered to precisely control and manipulate the properties of a light beam, such as its phase, amplitude, and polarization. Their evolution in the last two decades has enabled the miniaturization of numerous optical components, including holographic optical elements [1–3], beam deflectors [2, 4], and flat lenses [5–8]. More recently, reconfigurable metasurfaces have enabled active control of the optical properties revolutionizing the design, functionality and application domains of optical components and devices [9–12]. Phase-change materials [9, 12], [13], [14], stretchable substrates [15–17], strain-field engineering [18], thermo-optical effects [19–22], and free-carrier modulation [10, 11, 23, 24] are some of the engineering strategies used to achieve reversible changes of the metasurface function.

Reconfigurable (varifocal) metalenses (R-MLs) [14, 15, 20, 22, 24], in particular, have attracted increasing attention thanks to their enabling potential for tunable optics in microscopy systems [25], depth sensor devices [26, 27] as well as virtual and augmented reality platforms [28]. Moreover, these R-MLs can uniquely combine focal length modulation with complex functionalities, such as multi-wavelength [27] operation, spectroscopy and polarization routing [29], that are critical for compact and smart imaging devices/sensors.

**Figure 1:**
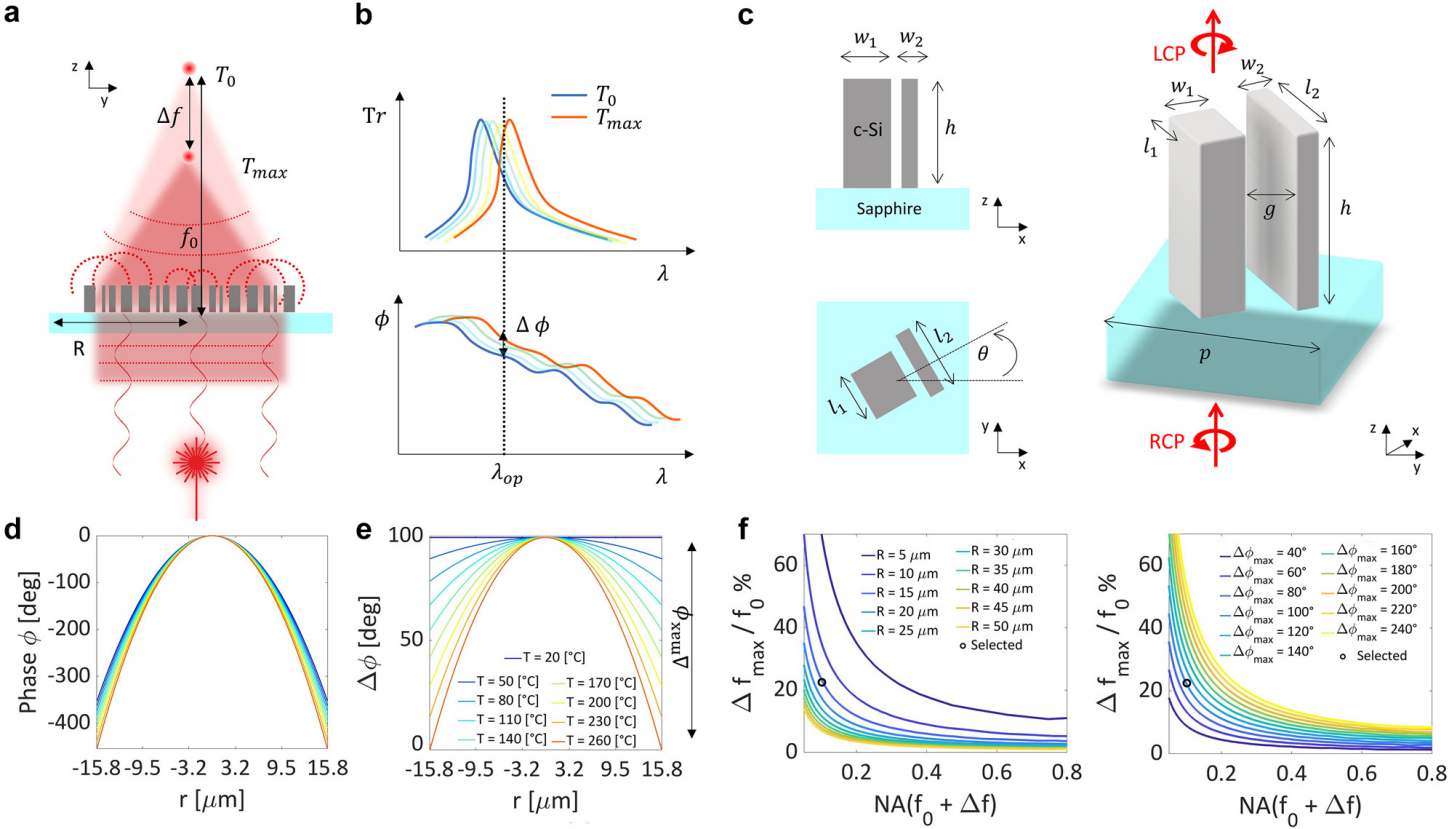
Thermally reconfigurable phase control: Principle and analytical phase design. (a) Schematic illustration of a thermally reconfigurable metalens. Each nano-structure exhibits a resonance mode that locally induces a phase delay. All together these meta-atoms shape a converging wavefront with focal length *f*_0_, which can be modulated with the temperature thanks to thermo-optical effects (schematic shows a decrease in focal length). (b) Schematic illustration of the thermo-optical effect. Thermally induced changes of the refractive index cause a shift of the resonance mode of a meta-atom (top). The associated change in phase delay results in a phase shift Δ*ϕ* at the operating wavelength *λ*_*op*_ (bottom). (c) Lateral and top views of a nanofin (left) as well as its 3D rendering, indicating also the illumination conditions (right). $\left(l_{1},w_{1}\right.$ , $\left.l_{2},w_{2}\right)$ , *gap*, *h*, *p* and *θ* are the width and the length of the nanopillar 1 and 2, the gap between the two nano-pillars, the height of the nano-pillars corresponding to the initial c-Si film thickness, the nano-fin lattice period and the angle of rotation of the nanofin structure respectively. *g* = 0.060 μm, *h* = 0.300 μm, *p* = 0.350 μm. (d) Required phase profile at different temperatures to satisfy: Initial focal length *f*_0_ = 200 μm at *T*_0_ = 20 °C; final focal length *f*_0_ + Δ*f* = 155 μm at *T*_max_ = 260 °C. (e) Required phase shift $\Delta \phi \left(r,T\right)$ between the phase profile at *T*_0_ = 20 °C and the phase at temperature *T*. (f) Numerical simulation of the maximum focal length variation over the initial focal length (%) as a function of the numerical aperture NA achievable with a maximum phase variation of Δ^max^ *ϕ* ∼ 100 deg and with different ML radiuses (left). Maximum focal length variation over the initial focal length (%) as a function of NA achievable with a ML radius *R* = 15.75 μm and for different Δ^max^ *ϕ* values (right). The chosen metalens design values are indicated with a black circle (*f*_0_ = 200 μm, Δ*f* = −45 μm and Δ^max^ *ϕ* = 102 deg).

The realization of *continuously* reconfigurable MLs, however, presents significant challenges. Indeed, to generate a converging wavefront with a desired focal length, the nano-scatters constituting a ML must introduce a prescribed (parabolic) phase delay at each position along the ML radius (Figure 1a–c). To dynamically change the focal length, a distinct phase shift must be achieved at each lattice site. Thus, R-MLs require the non-trivial realization of both a parabolic spatial phase-profile and a spatially varying phase shift (Figure 1d and e).

To date, stretchable substrates [30] and strain-field engineering [18], which entail a mechanical modification of the ML structure, have demonstrated excellent reconfiguration capabilities with good efficiency (up to 30 and 60% respectively) and large focal length tuning (up to 20 and 100% respectively). Yet, there is a growing interest for non-mechanical modulation approaches such as phase-change materials, thermo-optical effects [31] as well as tuning of free-carriers and exciton-resonances [32]. Indeed, these designs can offer unique opportunities in terms of fast modulation speed, including ultra-fast all-optical control [33], and reduced device thickness, i.e., ultra-thin lenses [34]. Furthermore, achieving the desired modulation using a single uniform external control of the local optical properties (e.g., temperature or electrical potential), would greatly simplify device design, fabrication and integration, accelerating the deployment in real-word components.

Thermo-optic effects represent an attractive approach for the realization of dielectric R-MLs [22]. Indeed, they entail a continuous and smooth change in the material optical properties (the refractive-index as shown in Supplementary Figure 1) and are robust against thermal-cycling. Yet, the low-magnitude of typical thermo-optical coefficients is generally assumed to limit the applicability of this strategy. Interestingly, silicon nano-resonators [5, 28] have been recently shown to exhibit pronounced shifts in their optical resonances (Figure 1b) both under external heating [19, 21] and for all-optical [35] modulation. Thus, by leveraging the amplification of thermo-optical effects by optical-resonance modes, silicon-based thermally reconfigurable dielectric metalenses (TR-MLs) could become a competitive and CMOS-compatible solution [36]. However, a viable design for such an ultra-thin, tunable ML with fixed operation wavelength in the visible regime has not been demonstrated yet.

Here we report a proof-of-concept design of an ultrathin (300 nm thick) and thermo-optically reconfigurable silicon ML operating at a fixed wavelength in the visible regime (632 nm). We demonstrate that, using thermo-optic effects, it is indeed possible to achieve continuous modulation of the focal-length beyond the depth-of-focus of the lens. Specifically, operating under right-circularly polarized light, our TR-ML exhibits a change of 21% in the focal length, with a linear shift from 165 μm at 20 °C to 135 μm at 260 °C. The average conversion efficiency of the lens is 26%, close to the performance of mechanically modulated devices, while its Strehl ratio is 0.99, confirming a diffraction-limited performance. Importantly, in our design, we rely on a spatially uniform temperature increase of the structure, overcoming the need for a spatially varying modulation input and potentially enabling an all-optical photo-thermal modulation approach. Concurrently, in this work, we report an automatized methodology to design a reconfigurable metalens, compute its layout and verify the expected performance. Overall, although further optimization of the meta-atom design is needed to boost the performance of these components [37], our results demonstrate that TR-MLs can be a viable solution for active tuning of optical systems.

## 2 Results

### 2.1 Thermally reconfigurable metalens phase profile and choice of design parameters

A TR-ML must be composed of an array of nano-scatters (meta-atoms – Figure 1a–c) that, at all temperatures *T*, satisfies a quadratic phase profile [38] along its radius *r*:

(1) $$\phi \left(r,f\left(T\right)\right)=-\frac{2\pi }{\lambda }\left[\sqrt{r^{2}+f^{2}\left(T\right)}-f\left(T\right)\right]$$

where $f\left(T\right)$ is the temperature-dependent focal length and *λ* the wavelength of the incident light. Fixing the desired focal length at the initial temperature *T*_0_, $f_{0}=f\left(T_{0}\right)$ , and requiring *f* to be linearly dependent on the temperature *T*, it is possible to express $f\left(T\right)$ as:

(2) $$\begin{array}{cc} f\left(T\right)=\left(T-T_{0}\right)\frac{\Delta f}{\Delta T}+f_{0} & \begin{array}{c} \Delta T=T_{\max}-T_{0}\\ \Delta f=f\left(T_{\max}\right)-f_{0} \end{array} \end{array}$$

where Δ*f* is the desired focal-length variation achievable at the maximum temperature variation Δ*T*. Using this assumption for $f\left(T\right)$ , and omitting the dependence on the fixed parameters *T*_0_, Δ*T*, *f*_0_, Δ*f*, the phase can be rewritten as follows:

(3) $$\phi \left(r,T\right)=\phi_{0}\left(r\right)+\Delta \phi \left(r,T\right)$$

where $\phi_{0}\left(r\right)=\phi \left(r,f_{0}\right)$ and the required phase shift at each temperature is:

(4) $$\Delta \phi \left(r,T\right)=\phi \left(r,T\right)-\frac{2\pi }{\lambda }\left[\sqrt{r^{2}+{f_{0}}^{2}}-f_{0}\right]$$

We observe that in Eqn. (3) the required temperature-dependent phase profile *ϕ*(*r*,*T*) has been decomposed into a temperature-independent initial phase profile, *ϕ*_0_(*r*), and a *spatially varying* phase shift, Δ*ϕ*, which depends on the temperature. Tuning of the phase shift at each lattice site can be achieved either by a structured external control [34] *T*(*r*) that generates different modulation inputs along the radius, i.e., $\Delta \phi \left(T\left(r\right)\right)$ , or by using meta-atoms that respond differently to the same uniform external control [20] *T*, i.e., $\Delta \phi \left(r,T\right)$ . In our system, the phase shift must be generated by thermo-optical effects in dielectric nanofins. Applying different temperatures to nano-structures that are closely spaced is very challenging. We thus pursue the second strategy, and we consider a spatially uniform temperature input *T* (Figure 1a).

The analytical phase profile described in Eqn. (3) is uniquely defined once *λ*, Δ*T*, *f*_0_, Δ*f*, and the ML radius *R* are defined. We note that for each set of these constraints, a specific phase shift range Δ^max^ *ϕ* must be attained with the meta-atoms (Figure 1c), where:

(5) $$\Delta^{\max}\phi =\max\left(\Delta \phi \left(r,T_{\max}\right)\right)-\min\left(\Delta \phi \left(r,T_{\max}\right)\right)$$

Seeking a favorable compromise between tunability and transmission efficiency of the ML in the visible regime, we set *λ* = 632 nm as the operating wavelength (see Supplementary Information S1, Figure 1b and Supplementary Figure 1 for further details). We also considered a maximum temperature variation Δ*T* = 240 °C, compatible with simple electric heaters (*T*_max_ = 260 °C). Next, we quantified Δ*f*/*f*_0_ as a function of the lens numerical aperture (NA), its radius *R* as well as Δ^max^ *ϕ* and we studied the maximum focal length variation versus the depth of focus (Figure 1f and Supplementary Figure 3). From this detailed analysis we observed that, in percentage, Δ*f*/*f*_0_ decreases for larger *R* and for smaller Δ^max^ *ϕ*. Aiming for a ML with a numerical aperture (NA) of at least 0.08, comparable with commercial ultra-compact objectives, and with a focal length variation at least equal to the depth of focus, we defined the target phase profile of the TR-ML using the following parameters: $f_{0}=f\left(T_{0}\right)=200\;\mu \text{m}$ , Δ*f* = −45 μm and *R* = 15.75 μm. We thus obtain NA(*f*_0_) = 0.08, NA(*f*_0_ + Δ*f*) = 0.10, Δ^max^ *ϕ* = 102 deg, the average depth of focus *z*_0_ ∼ 40 μm and the linear increase of the focal length with temperature is Δ*f*/Δ*T* ∼ 1.9 μm/°C. The ideal ML phase profile and required phase change obtained for the chosen set of parameters are shown in Figure 1e and f.

### 2.2 Metalens design: silicon nanofin library and digitalization of the target phase profile

The response of individual meta-atoms must be engineered such that their phase parameter space (*ϕ* and Δ*ϕ*) satisfies the requirements imposed by the target phase $\phi \left(r,T\right)$ and phase shift $\Delta \phi \left(r,T\right)$ . Specifically, based on our design parameters discussed above, we require a phase range of $\phi =\left[0,2\pi \right]$ , a maximum phase shift range of Δ^max^ *ϕ* ∼ 100 deg (Figure 1d and e) and a linear variation of the phase shift Δ*ϕ* with temperature (Supplementary Figure 2).

Non-resonant silicon nano-pillars, which only support waveguide-modes [5, 39, 40], are insufficient to realize an ultra-thin, sub-μm thick ML (*h* = 300 nm) because, for Δ*T* = 240 °C, they can achieve a maximum phase shift $\Delta^{\max}\phi ∼\frac{2\pi }{\lambda }h\Delta^{\max}n∼12{}^{\circ}\ll 100{}^{\circ}$ (see Supplementary Note S1, S3 and Supplementary Figure 1). Ultra-thin, resonant meta-atoms are instead expected to provide a wider phase space thanks to the amplification of thermo-optical effects by the different optical modes [19, 22, 35] (Figure 1b). Yet, simple geometries supporting a single resonance cannot produce a phase modulation range exceeding *π* [41].

To introduce additional degrees of freedom for engineering the phase profile, we therefore adopted anisotropic meta-atoms (nanofin) composed of two silicon nano-pillars waveguides with different nano-pillar length and width that act as coupled waveguides (Figure 1c). Indeed, these anisotropic nanofins offer the opportunity to leverage the Pancharatnam-Berry phase [42, 43] and obtain an additional *π* phase accumulation [44] (see Methods for further details).

**Figure 2:**
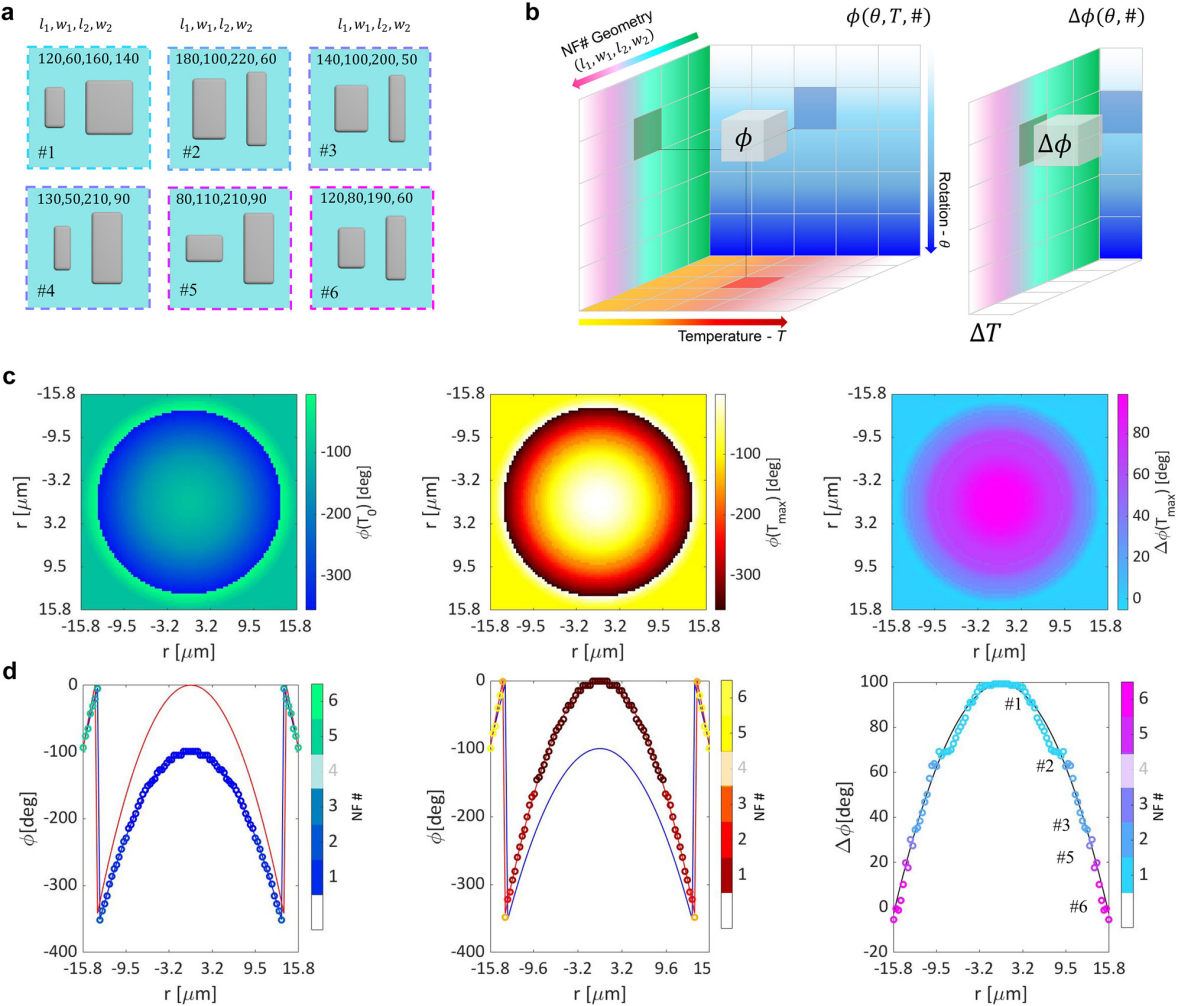
Study of the thermo-optic response of nanofins with different geometrical parameters and relative rotation. (a) Schematic illustration of a unit nanofin NF # structure and its geometrical parameters; $\left(l_{1},w_{1}\right.$ , $\left.l_{2},w_{2}\right)$ , *g*, *h*, *p* and *θ* are the width and the length of the nanopillar 1 and 2, the gap between the two nano-pillars, the height of the nano-pillars corresponding to the initial c-Si film thickness, the nano-fin lattice period and the angle of rotation of the nanofin structure respectively. *gap* = 0.060 μm, *h* = 0.300 μm, *p* = 0.350 μm. (b) *ϕ*(*θ*, *T*, *#*) and Δ*ϕ*(*θ*, *#*) are a 3D and 2D matrices representing the phase parameter space used for the wavefront design. (c) Left and center 2D metalens phase profile at 20 °C and 260 °C respectively. (c) Right metalens phase shift between 260° and 20 °C. (d) Left and center 1D projection of the 2D plots (c) left and center respectively. Circle markers represent the actual ML phase value at a specific radial position overlapped to the theoretical phase values displayed with a continuous line (blue and red color indicates 20 °C and 260 °C respectively. (d) Right 1D projection of the 2D plots in (c) right. Circle markers represent the actual ML phase shift value at a specific radial position overlapped to the theoretical phase shift values displayed with a continuous black line. Different gradual colors in plot (c) and (d) represent different nanofin (#1–6). Notice that the nanofin #4 has not been selected by the algorithm for the ML design. All the phase shift profiles are computed with respect to the initial temperature *T*_0_ = 20 °C. All the nanofin structures have been studied with COMSOL numeric simulations to retrieve their phase and transmission efficiency at different temperatures and rotation angles *θ*.

As shown in Figures 1c and 2a, the engineered nanofins are characterized by a set of geometrical parameters $\#=\left(l_{1},w_{1}\right.,$ $\left.l_{2},w_{2}\right)$ , where 1 and 2 indicate the first and the second nano-pillar, respectively, and by their rotation angle *θ*. The gap between the two nano-pillars (*g* = 60 nm) and their height (*h* = 300 nm) are fixed for all the nanofins (see Methods for further details). These are distributed with varying rotation angles in a sub-wavelength lattice on top of a sapphire substrate with a fixed period (*p* = 350 nm).

We used numerical simulations (COMSOL Multiphysics^®^, see Methods and Supplementary Note S3 for further details) to study the electromagnetic response of the nanofins. To obtain our nanofin library, we first performed a parameter sweep computing the transmission efficiency versus the maximum phase shift for every geometry # at a fixed angle *θ* = 0 and for Δ*T* = 240 °C. Interestingly, as shown in Supplementary Figure 14, there appears to be a trade-off between the maximum phase shift and the transmission efficiency. From this first study, we selected those geometries (Figure 2a) with an average transmission efficiency above 15% (Supplementary Table 1) that could also cover the required Δ^max^ *ϕ* ∼100 deg. The geometric parameters of these selected nanofins are shown in Supplementary Figure 6a and listed in Supplementary Table 1. Next, for every selected geometry #, we performed a sweep over the rotation angle *θ* and input temperature *T*, obtaining the complete dataset of phase $\phi \left(\theta ,T,\#\right)$ , phase shift Δ*ϕ*(*θ*, *#*) and transmission efficiency (Figure 2b, Supplementary Figure 4, Supplementary Figure 5). Finally, the nanofins must be distributed spatially based on the discretization of the analytical phase constraints. We thus constructed a routine that searched our dataset to identify, for every spatial position, the geometry (#) and rotation angle (*θ*) that satisfied the required phase-delay at both *T* = 20 °C and *T* = 260 °C as well as the linearity condition (Supplementary Note S2 and Supplementary Figure 2). With this approach we obtained the phase profile digitalization and the resulting layout for an optimal design of our TR-ML (Figure 2c and d, Supplementary Figure 5 and Supplementary Figure 6). In particular, we observe that, leveraging the PB phase, only five nanofin geometries are sufficient to satisfy well all the constraints.

Overall, we observed that the thermo-optical effect combined with the Pancharatnam-Berry (geometrical) phase effect in our engineered silicon nanofins provides a large range of phase and phase shift values enabling the design of a thermally reconfigurable metalens (TR-ML).

### 2.3 Thermally reconfigurable metalens

**Figure 3:**
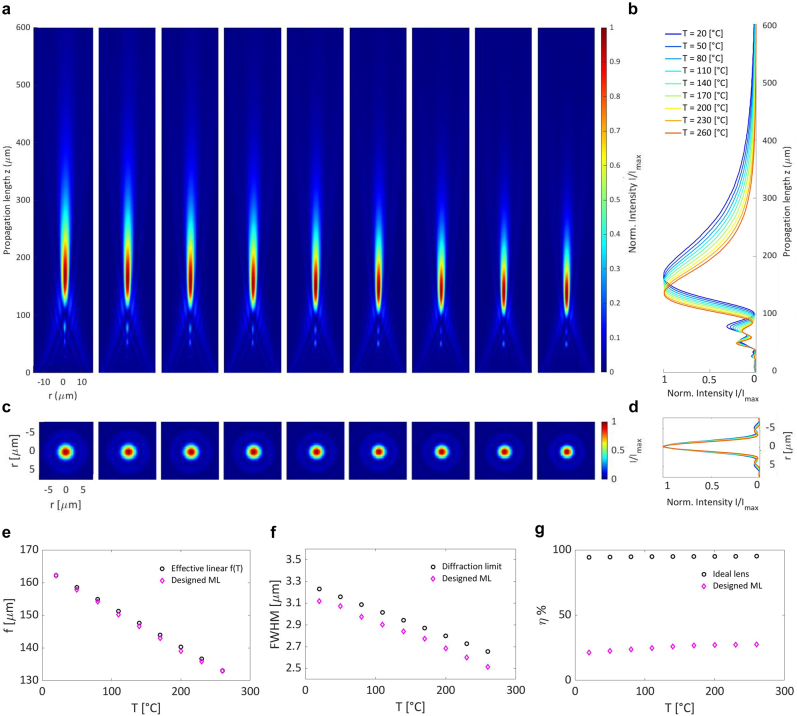
Thermally tunable metalens performance and deviation from an ideal lens. (a) Beam propagation focused by the designed ML. 2D intensity profiles along the XZ propagation plane at increasing temperatures. (b) 1D projection of the intensity profiles along the Z propagation direction at increasing temperatures. (c) Point spread function (PSF) 2D profile at the focal plane (XY) at increasing temperatures. (d) 1D projection of the PSF at all simulated temperatures. All the intensity profiles are normalized by the maximum intensity value at each temperature. (e) Focal length *f* at increasing temperatures. The focal length position of our designed metalens is compared with the focal length position extracted from the beam propagation of the ideal ML and with the effective linear focal length assumption. (f) PSF full width at half maximum (FWHM) of our designed ML at increasing temperatures. The FWHM of our designed ML is compared with the FWHM expected from an ideal ML and with the theoretical diffraction limit extracted from the Gaussian approximation of the Airy disk (*σ* ∼ 0.42*λ*/2*NA* and $\text{FWHM}=2\sqrt{2\;\ln\left(2\right)}*\sigma$ ). (g) Focusing efficiency *η* of our designed metalens at increasing temperature. *η* is computed as the transmitted field intensity over an integration area with radius *R*_*i*_ = 15.75 μm equal to the ML radius divided by the input field intensity at the ML plane (*z* = 0 μm). The average transmission efficiency of our designed ML over all temperature is 26%. The efficiency of our designed ML is compared with the efficiency expected by an ideal lens. With ideal lens we refer to a lens with an ideal phase profile and with 100% transmission efficiency. All the conversion efficiency values are computed with the same integration radius *R*_*i*_ = 15.75 μm.

We simulated the propagation of a beam focused by our TR-ML obtaining the three-dimensional intensity profile of the focused beam (see Methods). From a qualitative inspection of the intensity profiles along the propagation direction, we verified that the focal length decreases as the temperature increases (Figure 3a and b). The focal length shifts of the metalenses were determined by measuring their point spread functions at increasing temperatures (from *T* = 20 °C up to *T* = 260 °C*,* with Δ*T* = 30 °C) along the propagation direction (*z*-axis) with 1 μm resolution. Furthermore, we observed that both the point-spread-function (PSF) of the beam focus and the depth of focus become narrower at higher temperatures. Thus, the lens NA increases with temperature, as expected (Figure 3c and d, f). From a quantitative analysis, we obtained that the ML focal length changes from *f*(20 °C) ∼ 165 μm to *f*(260 °C) ∼ 135 μm.

Due to intrinsic diffraction of low Fresnel number lens [45], our ML, exhibits an unavoidable deviation of its focal length from the ideal focal length defined with geometric optics. In particular, the peak irradiance position *Z*_*p*_ derived from diffraction theory lies at an average distance *δ* = *Z*_*p*_ − *f* ∼ 27 μm from the target focal length *f* over all temperatures. Nonetheless, the reported design satisfies all the tunability requirements. Indeed, the total focal length modulation, Δ*f* ∼ 30 μm, is comparable to the final depth of focus, *z*_0_ ∼ 35 μm. Furthermore, we observe that both the point-spread-function (PSF) of the beam focus and the depth of focus become narrower at higher temperatures as the NA increases from NA(20 °C) = 0.10 to $\text{NA}\left(260{}^{\circ}\text{C}\right)=0.12$ (Figure 3e). In addition, the modulation follows the desired linear trend. In fact, from a linear fit of the focal length versus temperature we obtain a slope Δ*f*/Δ*T* ∼ 1.3 μm/°C, a root mean square error *RMSE* = 3.7 μm and an R-squared value *R* ^2^ = 0.99.

Importantly, from the deviation of the discretized phase profile from the analytical one (Supplementary Figure 5d and e), we obtain a ML Strehl ratio of *S* ∼ 0.99 > 0.8, indicative of a diffraction-limited behavior of our design (see Methods for further details). Indeed, as shown in Figure 3f, the value of the full-width at half maximum of the Airy disk at the focal plane ( $\text{FWHM}=2\sqrt{2\;\ln\left(2\right)}*\sigma$ ) lies at less than 0.1 μm from the diffraction-limit one (*σ* ∼ 0.42*λ*/2*NA* in the Gaussian approximation).

We also characterized our TR-ML by measuring the focusing efficiency of the focal spot under RCP incident light. We defined the focusing efficiency as the focal spot power divided by transmitted power through an aperture with the same radius as the designed metalens. Our TR-ML exhibits an average focusing efficiency equal to 26%. Although, this value is limited by the optical losses and reduced transmission (intrinsically limited up to 50% by the polarization conversion and by the conversion-efficiency of the nanofin meta-atoms Supplementary Table 1), our result is comparable to that of mechanically actuated reconfigurable metalenses [30, 46, 47].

## 3 Discussion

Similar to other adaptive optic approaches [46, 48], the TR-ML proposed in this work is affected by chromatic aberrations. Our metalens is designed to operate at one wavelength of *λ* = 0.632 μm with an effective focal length *f* ∼ 150 μm and an effective *NA* = 0.1. At this operating wavelength, focal length and NA, and assuming that the fractional change in the focal length is equal to the fractional change in the wavelength (Δ*f*/*f* = Δ*λ*/*λ*), the theoretical operation bandwidth [46, 49] should be $\Delta \lambda ∼\pi /4*\text{ln}(2)*\lambda^{2}/f{NA}^{2}∼270\text{nm}$ . However, a linear dependence of the phase on the wavelength might be not a good approximation because of the presence of resonance modes. Therefore, to ensure achromatic tunable metalens, different approaches recently demonstrated [8, 50] should be specifically studied and combined for phase dispersion control over continuous bandwidth. In this regard, we note that for ML designs based on purely resonant meta-atoms, effective thermal modulation and broad achromatic bandwidth rely on contrasting design requirements (high- and low- quality factor modes, respectively).

Our design principle enables also higher NA thermally tunable metalens. As the proof of concept, we show in Supplementary Figure 16 the beam propagation resulting from the design of a thermally tunable metalens with *NA* = 0.4, radius *R* = 4.55 μm and a total focal length shift of 10% (from 10 μm up to 11 μm). This design is based on the same nano-resonators set identified for our initial design and has been obtained following the same methodological approach. In general, the maximum focal length shift is limited by the tradeoff between the metalens NA and radius as well as the tradeoff between the metalens NA and the maximum thermal phase shift provided by the nano-resonators (Supplementary Figure 1 and Supplementary Figure 2). Such trade-off also impacts the design of MLs with larger diameters, as they require a large maximum phase shift. However, selecting longer operation wavelengths, the ML design present a less stringent trade-off between maximum focal length shift and NA (see Supplementary Figure 16).

In this regard, although the thermally tunable metalens presented here has a small sub-millimeter aperture, our methods can be easily adapted to generate metalens designs with higher diameters while keeping the exact same set of nanofin resonators and parameter space. However, to reach high quality design while increasing the ML radius it is recommended either to decrease the total focal length variation requirement or to extend the parameter space by introducing other geometries to cover the range of the total phase thermal shift that will be required while increasing the ML radius. In particular, one possibility to increase the maximum phase shift is to combine both positive and negative phase shifts of the meta-atoms. Moreover, it would be interesting to explore the use of a material with a higher thermo-optic coefficient, such as germanium [51, 52], and to operate at infrared (telecom) wavelengths.

Concerning the TR-ML realization and operation, we envision the possibility of using a ring micro-heater to provide a uniform temperature bias to the lens. Indeed, this approach in combination with thin substrates can result in excellent temperature uniformity across the lens (Supplementary Figure S17). Implementation of a micro-heater underneath the lens would likely provide faster response [20]. Yet, perturbations to the input polarization and optical losses could hinder the ML operation. More interestingly, we envision the possibility of leveraging optical heating for fast all-optical modulation of the metalens [35]. In this case, collective heating effects are expected to result in a non-uniform temperature profile across the radius of the ML. While these should be carefully assessed in the design phase, the proposed methodology is already capable of accounting for an arbitrary temperature profile. From the long-term stability point of view, adverse effects of thermal stresses (i.e., delamination) are not expected for the low temperatures considered for this analysis. However, much higher operation temperatures as well as thermal fatigue over many thermal cycles should be carefully assessed experimentally towards fast and reliable modulation.

## 4 Conclusions

Overall, we have demonstrated a thermally reconfigurable metalens based on thermo-optic effects in silicon nanofin resonators. Our design enables a continuous modulation of the focal length with close-to diffraction limited optical performance and competitive focusing efficiency. Our approach combines a geometrical phase approach with a temperature dependent phase shift to tune the lens properties using a spatially uniform temperature input. Increasing the complexity of the meta-atom geometry and introducing more resonance modes [53] would offer a broader parameter space to search for TR-ML designs with advanced optical properties (high NA, high efficiency). Thus, we envision that coupling our approach with machine-learning algorithms [54, 55] capable of identifying non-intuitive meta-atom shapes, which cover a large phase shift range with high conversion efficiencies, will significantly improve the performance of thermally reconfigurable silicon metalenses. More broadly, the proposed design approach, based on geometric phase combined with thermo-optical effects, is expected to offer inspiration for the future realization of a broad class of active metasurfaces within the emerging field of thermo-nanophotonics [36].

## 5 Methods

### 5.1 Strehl ratio and focusing efficiency

Strehl ratio is defined as the ratio between the square of the electric field amplitude at the center of the designed intensity profile and the square of the electric field amplitude at the center of the ideal point spread function (PSF) [56–58]. It is given by the following equation:

(6) $$S=\frac{I_{\text{ML}}\left(0,0\right)}{I_{\text{th}}\left(0,0\right)}={\left|\left\langle {\text{e}}^{\text{i}\phi }\right\rangle \right|}^{2}\approx {\text{e}}^{-\sigma^{2}}$$

where $\sigma =\sqrt{\left\langle {\left(\phi -\bar{\phi }\right)}^{2}\right\rangle }$ is the root mean square of the deviation of the designed phase from the theoretical phase. A lens is typically considered to correspond to diffraction limited performance if the Strehl ratio exceeds 0.8 [56–58].

The root mean square deviation of the designed ML phase profile from the analytical phase profile results to be *σ*[*ϕ*(*T*_0_)] = 2.5 deg at 20 °C and $\sigma \left[\phi \left(T_{\max}\right)\right]=4.3\;\deg$ at 260 °C (Supplementary Figure 5d and e). These values result in a Strehl ratio of *S* ∼ 0.99.

The efficiency is computed as the ratio between the total left circular polarized light intensity at the focal point upon conversion from right (RCP) to left (LCP) polarized light divided by the total light intensity (RCP plus LCP) which would be transmitted and focused assuming no losses due to absorption or reflection.

### 5.2 Simulation of the silicon nano-resonators

The geometry of all nano-resonators was simulated with a fixed gap between the two nano-pillars (*g* = 60 nm) and height (*h* = 300 nm). The gap has been minimized to maximize the coupling and its value has been chosen based on fabrication considerations such as the resolution of e-beam lithography. The nano-pillar high was fixed at about *h* ∼ *λ*/2 to ensure good mode coupling.

We simulated the interaction of circularly polarized light (electrical field ***E***) with our nano-scatterers to extract both the phase *ϕ* and the transmission *T* (coincident with the polarization conversion efficiency from RCP to LCP) of the electric field. We thus used the radio frequency (RF) module of COMSOL Multiphysics v5.5 to solve the Maxwell’s equation:

(7) $$\nabla \times \left(\nabla \times E\right)-k_{0}^{2}\varepsilon_{\text{r}}E=0$$

where $\varepsilon_{\text{r}}={\left(n-\text{i}k\right)}^{2}=n^{2}$ , *n* and k are the real and the imaginary part of the refractive index *n* respectively, *k*_0_ = 2*π*/*λ* is the wave number of free space and where only the constitutive relations for linear materials are considered.

Each silicon nanofins unit was simulated setting a periodic boundary condition along the transverse direction with respect to the propagation of light and a perfectly matched layer and input/output ports boundary conditions along the longitudinal direction.

We used the scattering parameter *S*_21_, measured from the eigenmode expansion of the electromagnetic field at the output port 2 (see Supplementary Figure 7), to extract both the transmission *T* and phase *ϕ* of the electric field converted from right to left circularly polarized light:

$$\begin{array}{c} \phi =-\arg\left(E^{\text{out}}\right)\\ T=\text{abs}\left(E^{\text{out}}\right) \end{array}$$

where

$$S_{21}=\sqrt{\frac{\text{Power delivered to Port }2}{\text{Power delivered to Port }1}}$$

and where the input RCP light incident on the input port 1 (*S*_1_, S-parameter of incident wave) under sapphire substrate, and the output LCP component (*S*_2_) at the exit port 2 are:

$$\begin{array}{c} \text{Port}\;1\to E^{\text{in,RCP}}=\left(\begin{array}{c} 1\\ i \end{array}\right)\\ \text{Port}\;2\to E^{\text{out,LCP}}=\left(\begin{array}{c} 1\\ -i \end{array}\right) \end{array}$$

Note that, while performing COMSOL simulation, we inverted both the sign of output phase and the input/output polarization definition compared to the ones used in the formalism described above for the phase profile design of our metalens. We are inverting the phase and polarization because in COMSOL the phase convention is based on:

(8) $$E^{\text{CM}}\left(x,y,z,\phi \right)=E_{0}\left(x,y,z\right){\text{e}}^{-\text{i}\phi }$$

Thus, while in our formalism an increase of the phase value indicates retardation (delay accumulation) in phase profile, in COMSOL, a phase increase introduces an electrical field anticipation.

### 5.3 Thermally tunable metalens design method

The design of our metalens (ML) is based on three main steps written in MATLAB.

In the first step, the software computes the maximum focal length variation and numerical aperture (NA) for a metalens characterized by the inputs set by the user. At this stage, the input data simulated in COMSOL are loaded and shown and the analytical phase profile based on the user input parameters is created and displayed. The metalens design optimization and the distribution of the different NF# geometries over the ML surface, are performed during the second step where the metalens layout is created and the Strehl ratio is computed. In the last step the Beam Propagation Method (BPM) is used to retrieve the actual ML focus profile and to extract the ML focus position, its FWHM and its depth of focus and its efficiency at each temperature. Lastly, the ML performance is compared with the behavior of an ideal lens. Further details can be found in the Supplementary note S1 and in the following paragraph.

### 5.4 Beam propagation method (BPM)

In this section, we present the derivation of the theory supporting the beam propagation method here implemented to study the behavior of a beam focused by our designed metalens [59, 60]. All numerical calculations of the intensity profiles were performed in MATLAB environment.

When considering a plane wave incident on a homogeneous, isotropic, and linear medium, the electromagnetic wave equations reduce to the Helmholtz equation for ***E***:

(9) $$\left(\nabla^{2}+k^{2}\right)E\left(x,y,z\right){\text{e}}^{\text{i}\phi }=0$$

In the paraxial limit, the two-dimensional Fourier transform of the electric field ***E*** transverse to the propagation axis Z and at a certain fixed position z (i.e., the ***E*** field in the z = 0 plane) can be written as:

(10) $$\hat{E}\left(k_{x},k_{y};z\right)=\frac{1}{4\pi^{2}}\overset{\infty }{\underset{-\infty }{\iint }}E\left(x,y,z\right){\text{e}}^{\text{i}\phi }{\text{e}}^{-\text{i}\left[k_{x}x+k_{x}x\right]}dx\;dy$$

where *k*_*x*_, *k*_*y*_ are the spatial frequencies coordinates of the Cartesian transverse coordinates *x* and *y* and *ϕ* the phase value in the $\left(x,y,z\right)$ position.

Then, the inverse Fourier transform becomes:

(11) $$E\left(x,y,z\right)=\overset{\infty }{\underset{-\infty }{\iint }}\hat{E}\left(k_{x},k_{y};z\right){\text{e}}^{\text{i}\left[k_{x}x+k_{x}x\right]}dk_{x}\;dk_{y}$$

Knowing that:

(12) $$k_{z}=\sqrt{\left(k^{2}-k_{x}^{2}-k_{y}^{2}\right)}$$

and replacing the ***E*** field expression in the Helmholtz equation with the Fourier representation just derived above, we find that the evolution of the field in the propagation direction *Z*, in the Fourier space corresponds to a multiplication for a factor ${\text{e}}^{\pm \text{i}k_{z}z}$ :

(13) $$\hat{E}\left(k_{x},k_{y};z\right)=\hat{E}\left(k_{x},k_{y};0\right){\text{e}}^{\pm \text{i}k_{z}z}$$

In other words, the Fourier spectrum of ***E***, in an arbitrary image plane at the z location, corresponds to the spectrum in the object plane (*z* = 0) multiply by the factor ${\text{e}}^{\pm \text{i}k_{z}z}$ where the sign ‘+’ describes a wave propagating the forward positive direction *z* > 0 and ‘− ’ sign refers to a wave propagating in the negative half-space *z* < 0. Replacing the expression (13) into (11) we can express the electrical field as:

(14) $$E\left(x,y,z\right)=\overset{\infty }{\underset{-\infty }{\iint }}\hat{E}\left(k_{x},k_{y};0\right){\text{e}}^{\text{i}\left[k_{x}x+k_{x}x\right]}{\text{e}}^{\pm \text{i}k_{z}z}\text{d}k_{x}\text{d}k_{y}$$

where

(15) $$\hat{E}\left(k_{x},k_{y};0\right)=\frac{1}{4\pi^{2}}\overset{\infty }{\underset{-\infty }{\iint }}E_{0}{\text{e}}^{\text{i}\phi }{\text{e}}^{-\text{i}\left[k_{x}x+k_{x}x\right]}dx\;dy$$

is the Fourier transform of the electrical field at the ML plane (*z* = 0) and *ϕ* and ***E***_0_ and are the phase and the electrical field amplitude of each nano-resonator at the position (*x*, *y*) on the *z* = 0 plane respectively. The intensity profile of the beam focused by the designed metalens can be finally retrieved from the squared of the electrical field propagation:

(16) $$I\left(x,y,z\right)={\left|E\left(x,y,z\right)\right|}^{2}$$

### 5.5 Geometrical phase

The phase delay induced by dielectric nano-structures on the incident light can be controlled by tuning either the refractive index, the relative rotation or the resonances of the nano-structures. Both on- and off-resonance based approaches are strictly related to the operating wavelength, the refractive index of the utilized materials and the geometry and location of the nano-antennas. On the contrary, the relative rotation of the nano-pillars introduces a geometrical phase delay which depends only on the geometrical asymmetry of the nano-structures and on the polarization of the incident light.

According to the Pancharatnam–Berry geometric effect [42, 43], circularly polarized light incident on a periodic layer consisting of subwavelength and anisotropic structures, with different orientations *θ* respect to the reference, is transmitted as the sum of two components: one with the same phase delay and the same handedness of the incident light and the other with a phase delay *ϕ* proportional to the rotation angle *θ* of the structure and with opposite handedness. In further detail, if the electric field incident on the nano-structure is circularly polarized, that is, $E^{\text{in}}=\left(\begin{array}{c} 1\\ \pm \text{i} \end{array}\right)$ , then the output electric field is given by [42, 43, 61], [62], [63]:

(17) $$E^{\text{out}}=M\cdot E^{\text{in}}=\frac{t_{\text{l}}+t_{\text{s}}}{2}\left(\begin{array}{c} 1\\ \pm \text{i} \end{array}\right)+\frac{t_{\text{l}}-t_{\text{s}}}{2}{\text{e}}^{\pm \text{i}2\theta }\left(\begin{array}{c} 1\\ \mp \text{i} \end{array}\right)$$

where the entire operation is represented by the matrix:

(18) $$M=R\left(-\theta \right)\left(\begin{array}{cc} t_{\text{l}} & 0\\ 0 & t_{\text{s}} \end{array}\right)R\left(\theta \right)$$

where *t*_l_ and *t*_s_ are the complex transmittance coefficients corresponding to an incident light linearly polarized along the long and the short axis of the nanofin respectively, *θ* is the nano-structure rotation angle with respect to its long axis in the rotation, $R=\left(\begin{array}{cc} \cos\theta & \sin\theta \\ -\sin\theta & \cos\theta \end{array}\right)$ is the rotation matrix and $\left(\begin{array}{c} 1\\ \mp i \end{array}\right)$ the eigenvectors associated to the eigenvalues *e* ^± *i*2*θ*^. The resulting phase delay of the light component with opposite handedness, thus, is *ϕ* = ±2*θ*.

The design of our ML relies on the electric field component converted from right-handed circularly polarized light $E^{\text{in,RCP}}=\left(\begin{array}{c} 1\\ -i \end{array}\right)$ to left-handed circularly polarized light $E^{\text{out,LCP}}=\frac{t_{\text{l}}-t_{\text{s}}}{2}{\text{e}}^{-\text{i}2\theta }\left(\begin{array}{c} 1\\ +i \end{array}\right)$ . The latter allows an average phase delay $\phi \left(\theta ,T,\#\right)∼-2\theta$ that can be exploited to cover the phase range needed for a thermally tunable focal length (Figure 2c, Supplementary Figure 6).

## Supplementary Material

Supplementary Material Details

## References

[1] Huang Y.-W., Chen W. T., Tsai W.-Y. (2015). Aluminum plasmonic multicolor meta-hologram. *Nano Lett.*.

[2] Sun S., Yang K.-Y., Wang C.-M. (2012). High-efficiency broadband Anomalous reflection by gradient meta-surfaces. *Nano Lett.*.

[3] Chen W. T., Yang K.-Y., Wang C.-M. (2014). High-efficiency broadband meta-hologram with polarization-controlled dual images. *Nano Lett.*.

[4] Kildishev A. V., Boltasseva A., Shalaev V. M. (2013). Planar photonics with metasurfaces. *Science*.

[5] Arbabi A., Horie Y., Ball A. J., Bagheri M., Faraon A. (2015). Subwavelength-thick lenses with high numerical apertures and large efficiency based on high-contrast transmitarrays. *Nat. Commun.*.

[6] Khorasaninejad M., Chen W. T., Devlin R. C. (2016). Metalenses at visible wavelengths: diffraction-limited focusing and subwavelength resolution imaging. *Science*.

[7] Wang S., Wu P. C., Su V.-C. (2018). A broadband achromatic metalens in the visible. *Nat. Nanotechnol.*.

[8] Chen W. T., Zhu A. Y., Sanjeev V. (2018). A broadband achromatic metalens for focusing and imaging in the visible. *Nat. Nanotechnol.*.

[9] Chu C. H., Tseng M. L., Chen J. (2016). Active dielectric metasurface based on phase-change medium. *Laser Photon. Rev.*.

[10] Huang Y.-W., Lee H. W. H., Sokhoyan R. (2016). Gate-tunable conducting oxide metasurfaces. *Nano Lett.*.

[11] Du X., Yan F.-P., Wang W. (2019). Graphene-embedded broadband tunable metamaterial absorber in terahertz band. *J. Opt.*.

[12] Mou N., Liu X., Wei T. (2020). Large-scale, low-cost, broadband and tunable perfect optical absorber based on phase-change material. *Nanoscale*.

[13] Leitis A., Heßler A., Wahl S. (2020). All-dielectric programmable huygens’ metasurfaces. *Adv. Funct. Mater.*.

[14] Wang Q., Rogers E. T. F., Gholipour B. (2016). Optically reconfigurable metasurfaces and photonic devices based on phase change materials. *Nat. Photonics*.

[15] Kamali S. M., Arbabi E., Arbabi A., Horie Y., Faraon A. (2016). Highly tunable elastic dielectric metasurface lenses. *Laser Photon. Rev.*.

[16] Ee H.-S., Agarwal R. (2016). Tunable metasurface and flat optical zoom lens on a stretchable substrate. *Nano Lett.*.

[17] Malek S. C., Ee H.-S., Agarwal R. (2017). Strain multiplexed metasurface holograms on a stretchable substrate. *Nano Lett.*.

[18] She A., Zhang S., Shian S., Clarke D. R., Capasso F. (2018). Adaptive metalenses with simultaneous electrical control of focal length, astigmatism, and shift. *Sci. Adv.*.

[19] Rahmani M., Xu L., Miroshnichenko A. E. (2017). Reversible thermal tuning of all-dielectric metasurfaces. *Adv. Funct. Mater.*.

[20] Afridi A., Canet-Ferrer J., Philippet L., Osmond J., Berto P., Quidant R. (2018). Electrically driven varifocal silicon metalens. *ACS Photonics*.

[21] Lewi T., Butakov N. A., Schuller J. A. (2019). Thermal tuning capabilities of semiconductor metasurface resonators. *Nanophotonics*.

[22] Iyer P. P., DeCrescent R. A., Lewi T., Antonellis N., Schuller J. A. (2018). Uniform thermo-optic tunability of dielectric metalenses. *Phys. Rev. Appl.*.

[23] Brar V. W., Sherrott M. C., Jang M. S. (2015). Electronic modulation of infrared radiation in graphene plasmonic resonators. *Nat. Commun.*.

[24] Ding P., Li Y., Shao L. (2018). Graphene aperture-based metalens for dynamic focusing of terahertz waves. *Opt Express*.

[25] Chen W. T., Zhu A. Y., Khorasaninejad M. (2017). Immersion meta-lenses at visible wavelengths for nanoscale imaging. *Nano Lett.*.

[26] Guo Q., Huang Y.-W., Alexander E. (2019). Compact single-shot metalens depth sensors inspired by eyes of jumping spiders. *Proc. Natl. Acad. Sci.*.

[27] Lin R. J., Su V.-C., Wang S. (2019). Achromatic metalens array for full-colour light-field imaging. *Nat. Nanotechnol.*.

[28] Lee G.-Y., Hong J.-Y., Hwang S. (2018). Metasurface eyepiece for augmented reality. *Nat. Commun.*.

[29] Engelberg J., Levy U. (2020). The advantages of metalenses over diffractive lenses. *Nat. Commun.*.

[30] Wei S., Cao G., Lin H. (2021). A varifocal graphene metalens for broadband zoom imaging covering the entire visible region. *ACS Nano*.

[31] Zograf G. P., Petrov M. I., Makarov S. V., Kivshar Y. S. (2021). All-dielectric thermonanophotonics. ..

[32] van de Groep J., Song J.-H., Celano U. (2020). Exciton resonance tuning of an atomically thin lens. *Nat. Photonics*.

[33] Dias E. J. C., Yu R., García de Abajo F. J. (2020). Thermal manipulation of plasmons in atomically thin films. *Light Sci. Appl.*.

[34] Park S., Lee G., Park B. (2020). Electrically focus-tuneable ultrathin lens for high-resolution square subpixels. *Light Sci. Appl.*.

[35] Tsoulos T. V., Tagliabue G. (2020). Self-induced thermo-optical effects in silicon and germanium dielectric nanoresonators. *Nanophotonics*.

[36] Zograf G. P., Petrov M. I., Makarov S. V. (2021). All-dielectric thermonanophotonics. *Adv. Opt. Photonics*.

[37] Wang F., Geng G., Wang X. (2022). Visible achromatic metalens design based on artificial neural network. *Adv. Opt. Mater.*.

[38] Chen W. T., Zhu A. Y., Capasso F. (2020). Flat optics with dispersion-engineered metasurfaces. *Nat. Rev. Mater.*.

[39] Liu M., Fan Q., Yu L., Xu T. (2019). Polarization-independent infrared micro-lens array based on all-silicon metasurfaces. *Opt Express*.

[40] Lu X., Guo Y., Pu M. (2021). Broadband achromatic metasurfaces for sub-diffraction focusing in the visible. *Opt Express*.

[41] Chen M. K., Wu Y., Feng L. (2021). Principles, functions, and applications of optical meta-lens. *Adv. Opt. Mater.*.

[42] Gori F. (1999). Measuring Stokes parameters by means of a polarization grating. *Opt. Lett.*.

[43] Roux F. S. (2006). Geometric phase lens. *J. Opt. Soc. Am. A*.

[44] Chen W. T., Zhu A. Y., Sisler J., Bharwani Z., Capasso F. (2019). A broadband achromatic polarization-insensitive metalens consisting of anisotropic nanostructures. *Nat. Commun.*.

[45] Ruffieux P., Scharf T., Herzig H. P., Völkel R., Weible K. J. (2006). On the chromatic aberration of microlenses. *Opt Express*.

[46] Arbabi E., Arbabi A., Kamali S. M. (2018). MEMS-tunable dielectric metasurface lens. *Nat. Commun.*.

[47] Bosch M., Shcherbakov M. R., Won K., Lee H.-S., Kim Y., Shvets G. (2021). Electrically actuated varifocal lens based on liquid-crystal-embedded dielectric metasurfaces. *Nano Lett.*.

[48] Klopfer E., Lawrence M., Barton D. R., Dixon J., Dionne J. A. (2020). Dynamic focusing with high-quality-factor metalenses. *Nano Lett.*.

[49] Arbabi A., Arbabi E., Kamali S. M. (2016). Miniature optical planar camera based on a wide-angle metasurface doublet corrected for monochromatic aberrations. *Nat. Commun.*.

[50] Zhu A. Y., Chen W. T., Sisler J. (2019). Compact aberration-corrected spectrometers in the visible using dispersion-tailored metasurfaces. *Adv. Opt. Mater.*.

[51] Bosch M., Shcherbakov M. R., Fan Z., Shvets G. (2019). Polarization states synthesizer based on a thermo-optic dielectric metasurface. *J. Appl. Phys.*.

[52] Viña L., Logothetidis S., Cardona M. (1984). Temperature dependence of the dielectric function of germanium. *Phys. Rev. B*.

[53] Hsiao H.-H., Chen Y. H., Lin R. J. (2018). Integrated resonant unit of metasurfaces for broadband efficiency and phase manipulation. *Adv. Opt. Mater.*.

[54] An S., Zheng B., Shalaginov M. Y. (2020). Deep learning modeling approach for metasurfaces with high degrees of freedom. *Opt Express*.

[55] Zhelyeznyakov M. V., Brunton S. L., Majumdar A. (2020). Deep learning to accelerate Maxwell’s equations for inverse design of dielectric metasurfaces. *ArXiv200810632 Phys.*.

[56] Ottevaere H., Thienpont H., Guenther R. D. (2005). Optical microlenses. *Encyclopedia of Modern Optics*.

[57] Chuang K. L., Statistical K. K. (1986). *Analyis of the 70 Meter Antenna Surface Distorsion*.

[58] Mahajan V. N. (1983). Strehl ratio for primary aberrations in terms of their aberration variance. *JOSA*.

[59] Kamilov U. S., Papadopoulos I. N., Shoreh M. H. (2016). Optical tomographic image reconstruction based on beam propagation and sparse regularization. *IEEE Trans. Comput. Imaging*.

[60] Feit M. D., Fleck J. A. (1988). Beam nonparaxiality, filament formation, and beam breakup in the self-focusing of optical beams. *JOSA B*.

[61] Pancharatnam S. (1956). Generalized theory of interference, and its applications. *Proc. Indian Acad. Sci. Sect. A*.

[62] Bomzon Z., Biener G., Kleiner V., Hasman E. (2002). Space-variant Pancharatnam–Berry phase optical elements with computer-generated subwavelength gratings. *Opt. Lett.*.

[63] Zhan T., Xiong J., Lee Y.-H., Wu S.-T. (2018). Polarization-independent Pancharatnam-Berry phase lens system. *Opt Express*.

