# Supplementary material for: Thermally reconfigurable metalens
Source: Nanophotonics. 2022 May 30;11(17):3969–80. doi: 10.1515/nanoph-2022-0147 (PMC9394514; doi:10.1515/nanoph-2022-0147)
Supplement: Supplementary file 1 — Supplementary Material Details [file j_nanoph-2022-0147_suppl.pdf]

Supplementary Material for Thermally-reconfigurable metalens

Anna Archetti[1]†, *Ren-Jie* Lin[1]†, *Nathanael* Restori[1], *Fatemeh* Kiani[1], *Ted V.* Tsoulos[1], *Giulia* Tagliabue[1,*]

[1]Laboratory of Nanoscience for Energy Technologies (LNET), STI, École Polytechnique Fédérale de Lausanne (EPFL), 1015 Lausanne, Switzerland

† equal contribution

* corresponding author: giulia.tagliabue@epfl.ch

Supplementary Information

S1. Thermo-optic property of silicon

The design of our thermally tunable metalens relies on the variation of the refractive index of our silicon nanofins with temperature. An intuitive way to describe their optical properties is to treat them as truncated waveguide[1,2]. The effective refractive index n_{eff} of the nanofins affects the light propagating through them imparting a phase variation with temperature T which is different for different nanofin structure as follows: $\Delta\phi = \frac{2\pi}{\lambda}\Delta n_{eff}(\theta,\ T,\ \#)\, h,$ where h is the nanofin height, λ the operating wavelength, $\# = (l_1,\ w_1, l_2,\ w_2)$ the nanofin geometry and θ the nanofin rotation. On that account, each nanofin structure can locally induce both a different phase delay and a different phase variation with temperature along the ML radius to enables a spherical wavefront tunable with temperature control.

In terms of thermo-optical response, crystalline silicon is a promising material thanks to its large thermo-optical coefficient[3]. However, for wavelengths shorter than $\sim 600\ nm$, it exhibits significant optical losses (i.e. light absorption) that also increase with temperature (**Supplementary Figure 1c**). Indeed, both the real and imaginary parts n and k of the refractive index $n(T) = \mathrm{n}(T) + i\mathrm{k}(T)$ of silicon decrease as increasing the temperature T. Since the transmitted electrical field is affected by the value of extinction coefficient $\mathrm{k}(T)$ via the attenuation factor $e^{-\frac{2\pi}{\lambda}\mathrm{k}(T)\, h}$, its attenuation is higher at higher temperature:

$$E_z = E_0 e^{ik\, h} = E_0 e^{i\frac{2\pi}{\lambda} n(T)\, h} \tag{1}$$

$$E_z = E_0 e^{-\frac{2\pi}{\lambda}\mathrm{k}(T)\, h} e^{i\frac{2\pi}{\lambda}\mathrm{n}(T)\, h} \tag{2}$$

where with E_z is the electrical field component in the z direction at the center of the XY plane, ω is the angular frequency and k the wavenumber. Therefore, we select 632 nm as operation wavelength of metalens to prevent the higher loss in shorter wavelength. However, with non-resonant silicon nanofins, the maximum phase variation achievable with our maximum temperature shift $\Delta T(T_{\max} = 260°C, T_0 = 20°C) = 240°C$ at the operating wavelength $\lambda = 0.632\ \mu\mathrm{m}$ is $\Delta^{\max}\phi \sim \frac{2\pi}{\lambda} h\, \Delta^{\max} n \sim 12\ \mathrm{deg}$ where $\Delta^{\max} n$ is the maximum variation of the refractive index of silicon is $\Delta^{\max} n \sim 0.07$ with ΔT (**Supplementary Figure 1**). Therefore, we engineered the geometries of our nanofin to achieve higher phase variation via resonant modes (see **Supplementary Note S3 and S4**).

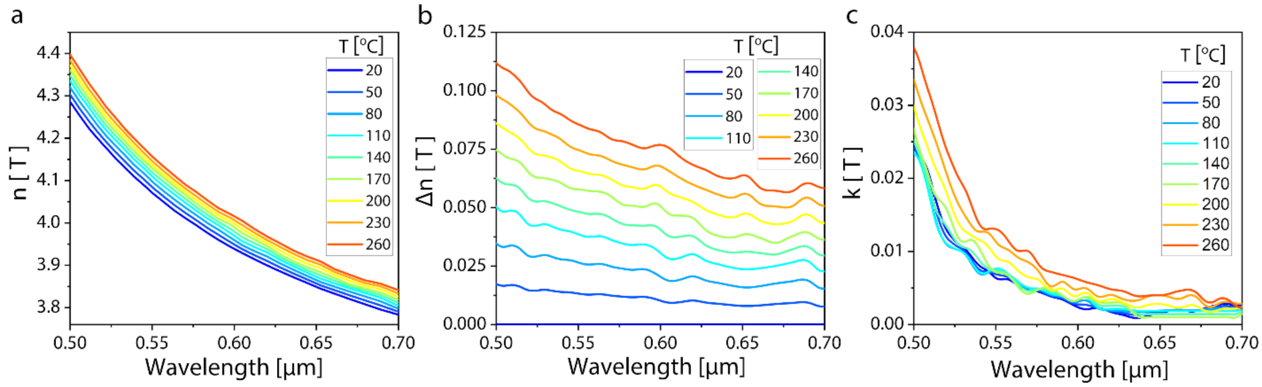

Supplementary Figure 1 – Temperature Dependence of the Optical Properties of Silicon. a Refractive index of Silicon as a function of the wavelength at different temperatures[3]. **b** Refractive index difference between the refractive index at temperature T and the refractive index at $T_0 = 20°C$. **c** Extinction coefficient (imaginary part coefficient of the refractive index). Variation relative to the concomitant**.**

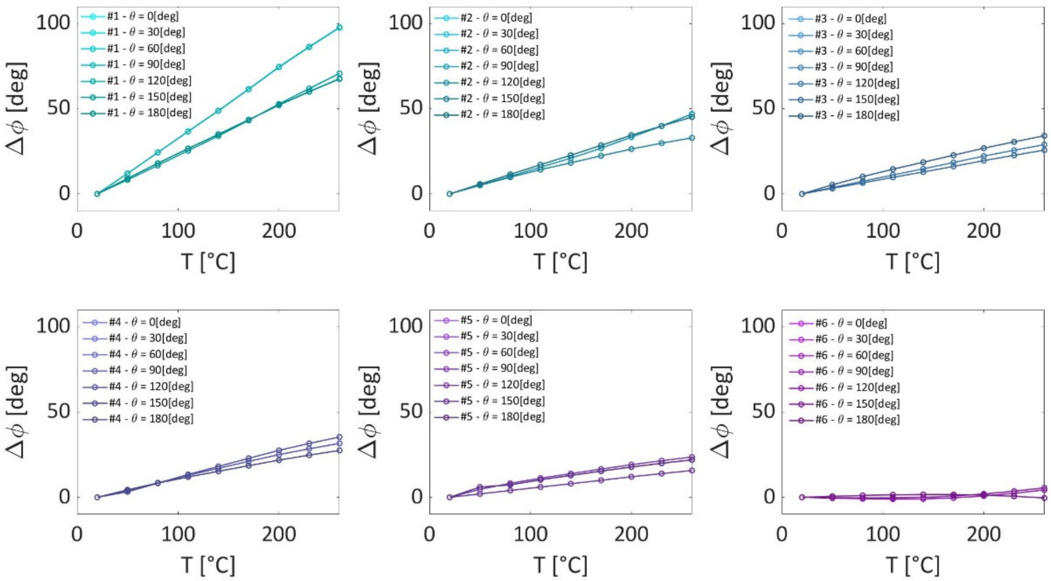

Supplementary Figure 2 – Thermal phase shift linearity. Phase shift as a function of temperature for different rotation angle for each nanofin. Color from blue to violet indicate different nanofin geometries.

S2. Verifocal metalens design: software pipeline

Below we report the eight main steps which we follow for the design of our metalens. All the MATLAB code is available upon request.

Step 1: Compute the maximum focal length variation and numerical aperture (NA) of a metalens characterized by the following inputs: a 3D phase matrix $\phi(\theta,\ T,\ \#)$, with the nano-resonator phase as function of three variables (angle θ, temperature T and nanofin geometry # in our case), the three independent variable ranges and the ML radius R. Compute the maximum phase variation and the numerical aperture (NA) of a metalens with the focal length variation Δf, the initial focal length f_0, and radius R chosen by the user.

Step 2: Create and show the target analytical phase profile based on the user inputs.

Step 3: Display the phase, transmission and phase variation inputs simulated in COMSOL.

Step 4: Metalens design: 1D optimization and distribution of the NF# geometries and angles

Step 5: Metalens 2D optimization and layout creation.

Step 6: Plot the metalens 2D layout plot and compute the Strehl ratio.

Step 7: Use the BSM Beam Propagation Method to retrieve the focus profile of the designed ML and of an ideal ML.

Step 8: Extract the focus position, its FWHM and depth of focus at each temperature of the designed ML and of an ideal ML.

Step 9: Compute the ML efficiency at each temperature.

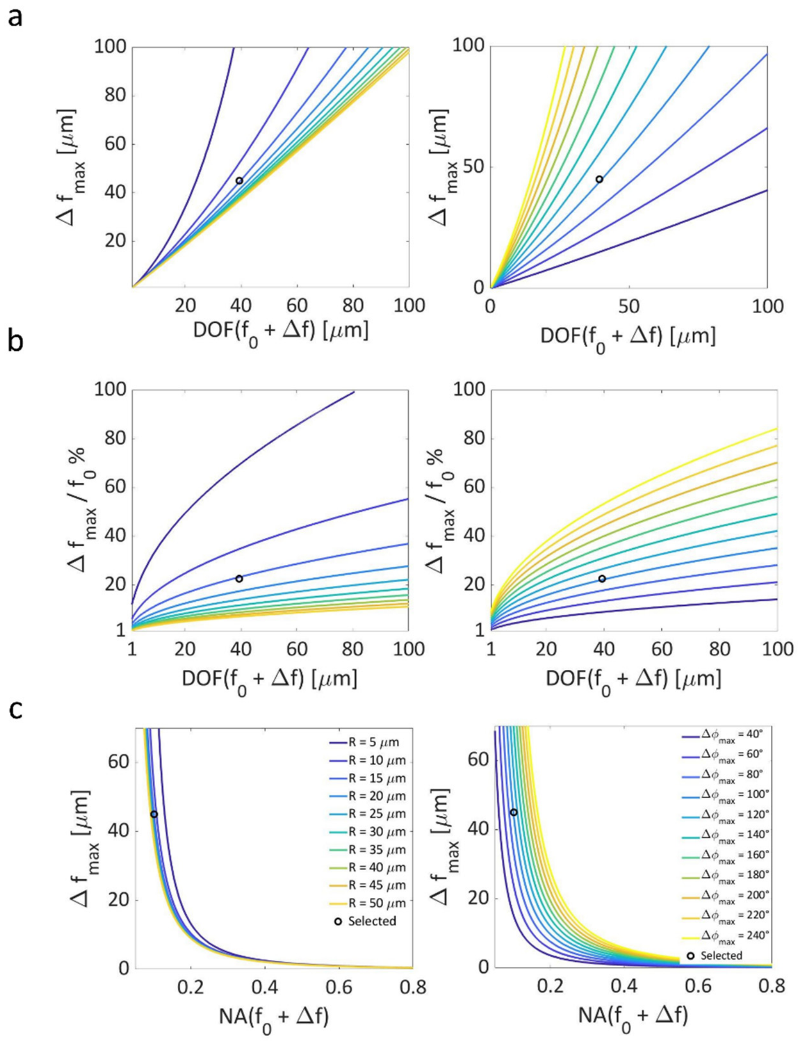

Supplementary Figure 3 – Numerical simulation of the maximum variation of the focal length versus the depth of focus ($\boldsymbol{DOF}$) and the numerical aperture (NA). a left Numerical simulation of the maximum focal length variation as a function of the DOF achievable with a maximum phase variation of $\Delta^{\max}\phi = 100\ \mathrm{deg}$ and with different ML radiuses; **a right** maximum focal length variation as a function of the DOF achievable with a ML radius $R = 15.75\ \mu m$ and with $\Delta^{\max}\phi$ values. **b left** Numerical simulation of the maximum focal length variation over the initial focal length (%) as a function of the DOF achievable with a maximum phase variation of $\Delta^{\max}\phi = 100\ \mathrm{deg}$ and with different ML radiuses; **b right** maximum focal length variation over the initial focal length as a function of the DOF achievable with a ML radius $R = 15.75\ \mu m$ and with $\Delta^{\max}\phi$ values. **c left** Numerical simulation of the maximum focal length variation as a function of the NA achievable with a maximum phase variation of $\Delta^{\max}\phi = 100\ \mathrm{deg}$ and with different ML radiuses; **c right** maximum focal length variation as a function of the NA achievable with a ML radius $R = 15.75\ \mu m$ and with $\Delta^{\max}\phi$ values. The selected value is obtained imposing $f_0 = 200\ \mu m, \Delta f = -45\ \mu m$ and $\Delta^{\max}\phi = 102\ \mathrm{deg}$

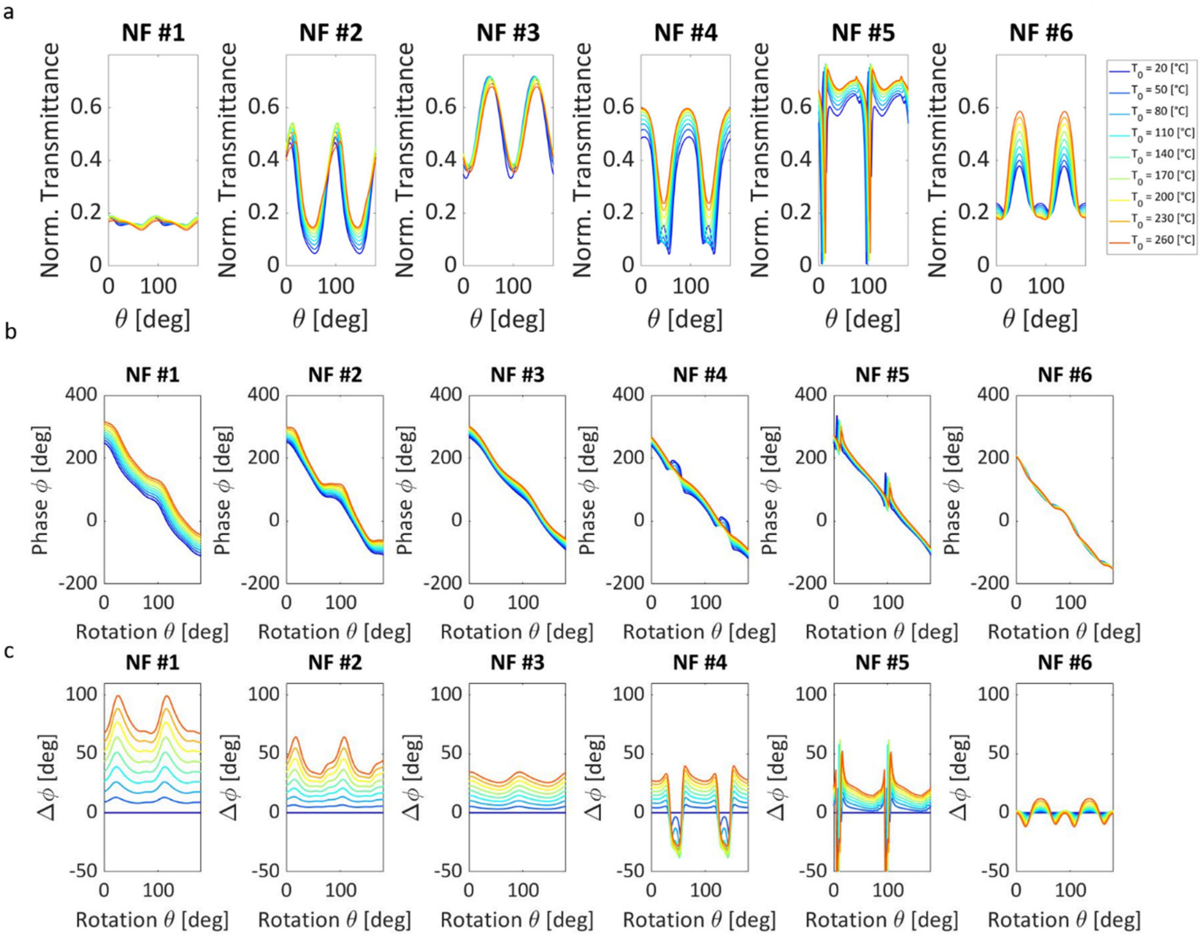

Supplementary Figure 4 – Nano-resonator COMSOL simulation results. Transmittance and phase value versus rotation angle. a Transmittance profiles as a function of the rotation angles θ for every nanofins geometries (NF #1-6) at increasing temperature (from 20°C to 260°C). **b and c** Temperature-dependent phase and phase variation profiles as a function of the rotation angles θ for every nanofins geometries (NF #1-6). All the phase variation profiles are computed with respect to the initial temperature $T_0 = 20°C$. All the nanofin structures have been studied with COMSOL numeric simulations to retrieve their phase and transmission efficiency at different temperatures and rotation angles θ. Colors from blue to red indicate an increase in temperature from 20°C up to 260°C with 30°C step size.

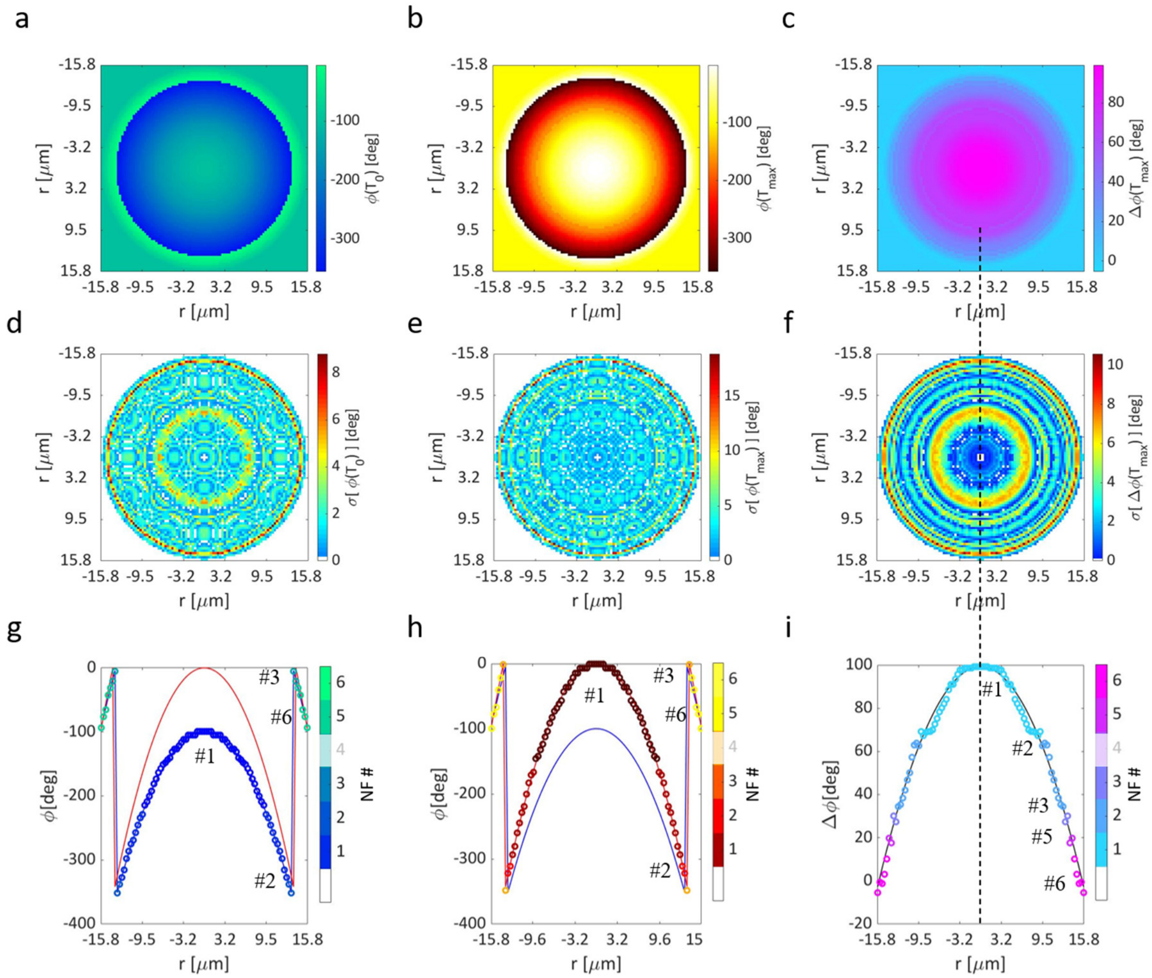

Supplementary Figure 5 – Metalens phase profile design. 1D and 2D visualization of the ML design quality compared to theoretical values. a and **b** 2D metalens phase profile at 20°C and 260°C respectively. **c** Metalens phase shift between 260° and 20°C. **d** and **e** 2D phase value error: distance from the theoretical value at 20°C and 260°C respectively. **f** Phase shift error: distance from the theoretical value. **g** and **h** 1D projection of the 2D plots **a** and **b** respectively. Circle markers represent the actual ML phase value at a specific radial position overlapped to the theoretical phase values displayed with a continuous line (blue and red color indicates 20°C and 260°C respectively. **i** 1D projection of the 2D plots in **c.** Circle markers represent the actual ML phase shift value at a specific radial position overlapped to the theoretical phase shift values displayed with a continuous black line. Different gradual colors in plot **g, h** and **i** represent different nanofin (#1-6). Notice that the nanofin #4 has not been selected by the algorithm for the ML design.

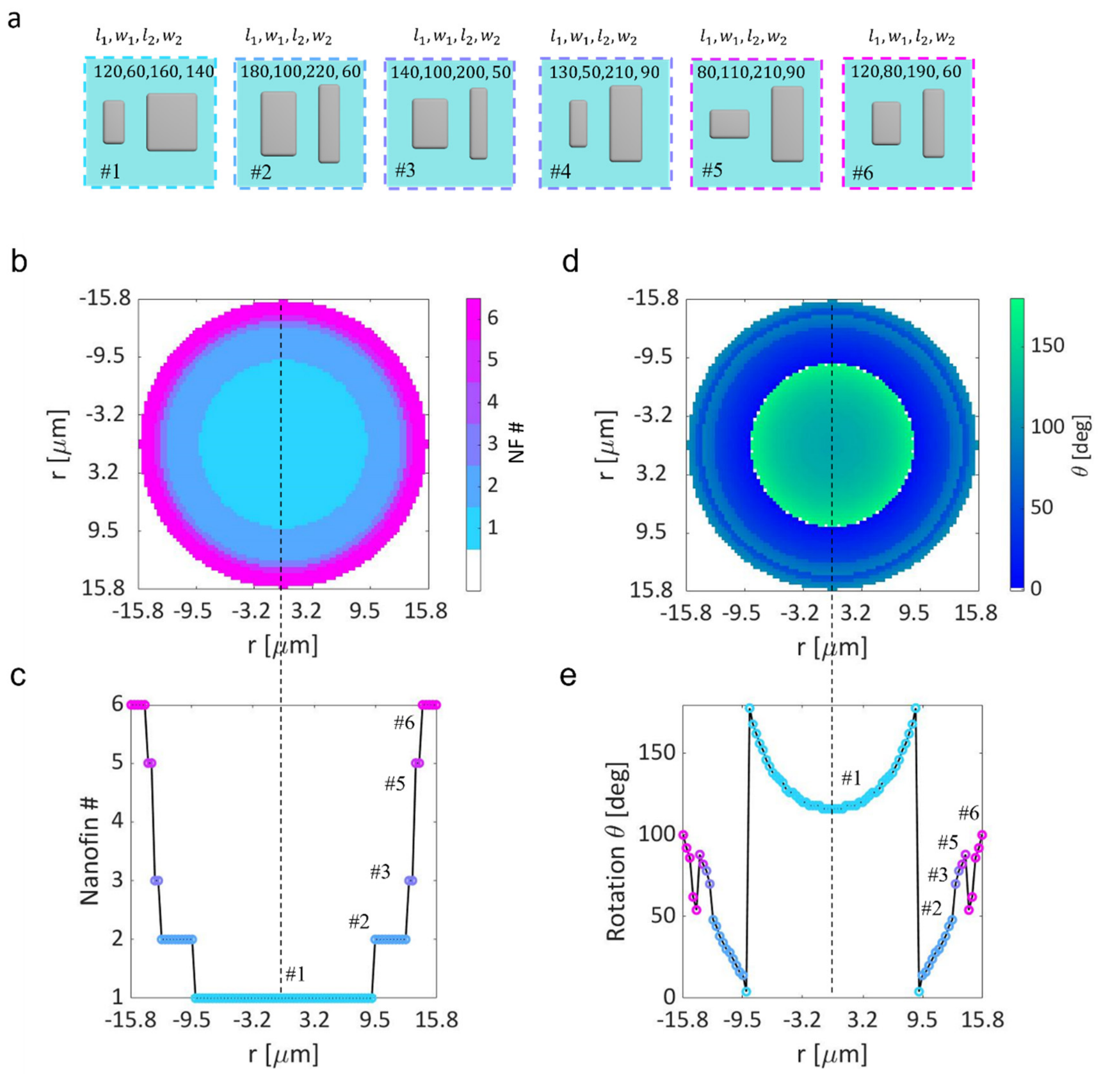

Supplementary Figure 6 – Thermally tunable metalens layout. a Nanofin selected geometry; **b** 2D distribution of the nanofin geometries over the metalens surface; **c** 1D distribution of the nanofin geometries along the metalens diameter; **d** 2D distribution of the nanofin angle over the metalens surface; **e** 1D distribution of the nano-fin angles along the metalens diameter. Color variation from pick to blue indicate different nanofin geometries (from #1 to #6).

#	$\Delta\phi$ [deg]	$\phi(T_0)$ [deg]	$Tr(T_0)$ %	θ range [deg]	θ_0 [deg]	θ_{max} [deg]	l_1 [nm]	w_1 [nm]	l_2 [nm]	w_2 [nm]	g [nm]	h [nm]
**6**	~ - 1	-191	24%	50 – 100	50	46	120	80	190	60	60	300
**5**	~ 20	-147	54%	82 – 88	88	106	80	110	210	90		
**4**	~ 30	-158	48%	x	x	x	130	50	210	90		
**3**	~ 30	-130	35%	70 – 80	78	88	140	100	200	50		
**2**	~ 48	-146	41%	12 – 50	12	12	180	100	220	60		
**1**	~ 80	-149	17%	116 – 180	116	96	120	60	160	140		

Supplementary Table 1 – Nanofin main parameters. Phase variation $\Delta\phi$ obtained with the largest temperature variation between $T_{\max} = 260°C$ and $T_0 = 20°C$ at the angle with highest transmittance among all the angles used for the ML design. Phase $\phi(T_0)$ and transmission efficiency $Tr(T_0)$ of the transmitted electrical field component with opposite handedness polarization compared to the incident one. Nanofin geometrical parameters # $= (l_1,\ w_1,\ l_2,\ w_2)$: where w_1 and w_2 are the widths and l_1 and l_2 the lengths of the first and second nano-pillar respectively. $\boldsymbol{\theta_0}$ is the angle with the highest average phase shift among all the angles used in the design. $\boldsymbol{\theta_{max}}$ is the angle with the highest average phase shift among all the angles.

S3. Numerical simulation of the silicon nanofins field

To study the nanofin thermal phase shift, we simulated the transmittance (polarization conversion efficiency) and phase spectra at 20°C and 260°C for each nanofin at all angles. For further detail see **Supplementary Table 1**, **Supplementary Figure 8**, **Supplementary Figure 9**, **Supplementary Figure 10**, **Supplementary Figure 11**, **Supplementary Figure 12**, **Supplementary Figure 13**.

To plot the distribution of conversion efficiency versus phase difference at 400° Δϕ(400°C), we paramedic sweep the parameters l_1, w_1, l_2, w_2 with 350 nm period p and 60 nm gap g as illustrated in **Figure 1c**. Each dot of **Supplementary Figure 14** represents an element with the specified parameter. The phase shift of each element is mainly distributed in the range of 10 to 70 deg with reasonable polarization conversion efficiency (transmittance) since mostly of elements are weak resonance or non-resonance in operation wavelength 632 nm.

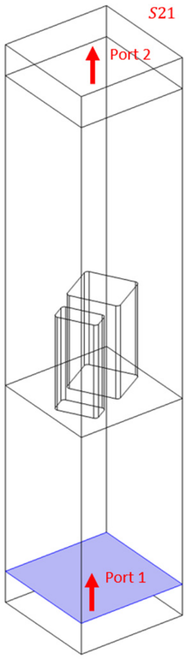

Supplementary Figure 7 – COMSOL simulation input and output ports. Port 1 is the input port at the interface between sapphire substrate and air. Port 2 is the output port on the top of the simulated air volume surrounding the silicon (Si) nanofin structure. Right hand circularly polarized light (RCP) is input at Port 1. Left hand circularly polarized light (LCP) is measured at Port 2 via the scattering parameter S_{21}.

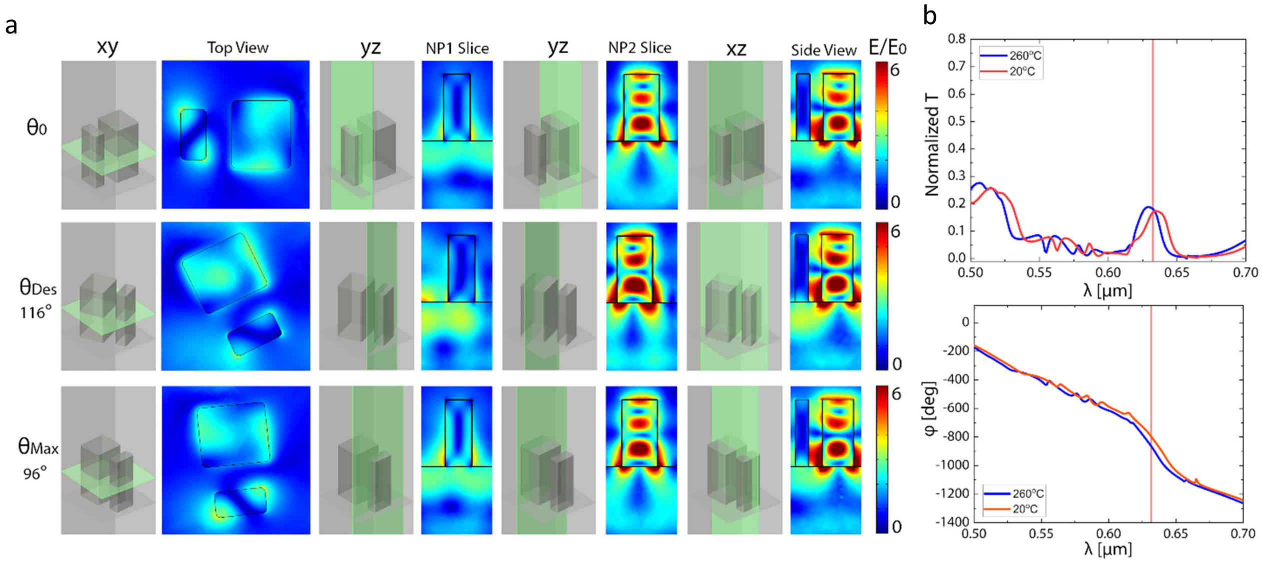

Supplementary Figure 8 – Nanofin #1. Numerical simulation. Electric field profile, transmittance, and phase. **a** Electric field for top view, side view of left nanopillar, side view of right nanopillar and side view of both pillar from left to right panels respectively. **b** Transmittance and phase spectra for the nanofin #1. at temperatures of 20°C and 260°C. $\boldsymbol{\theta_{Des}}$ is the angle with the highest average phase shift among all the angles used in the design. $\boldsymbol{\theta_{max}}$ is the angle with the highest average phase shift among all the angles.

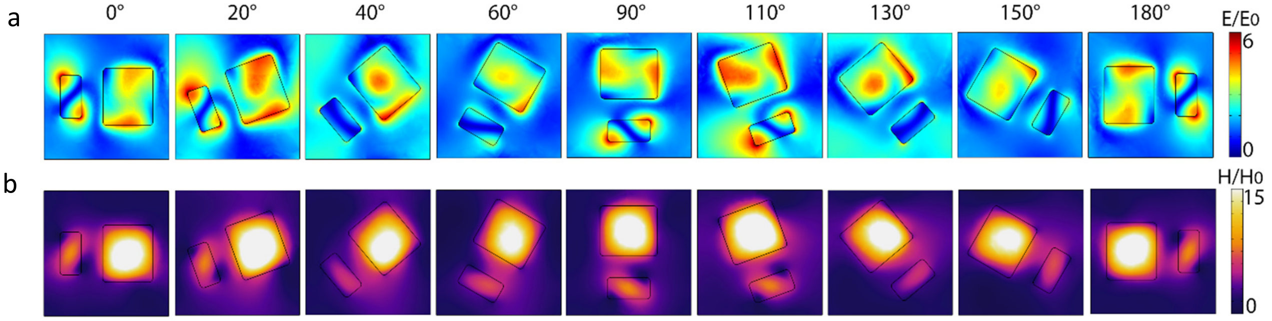

Supplementary Figure 9 – Nanofin #1. 2D electric and magnetic field profile at increasing rotation angle. **a** Electric filed for top view at increasing angles from 0 deg up to 180 deg for the nanofin #1. **b** Magnetic field from top view at increasing angles from 0 deg up to 180 deg for the nanofin #1.

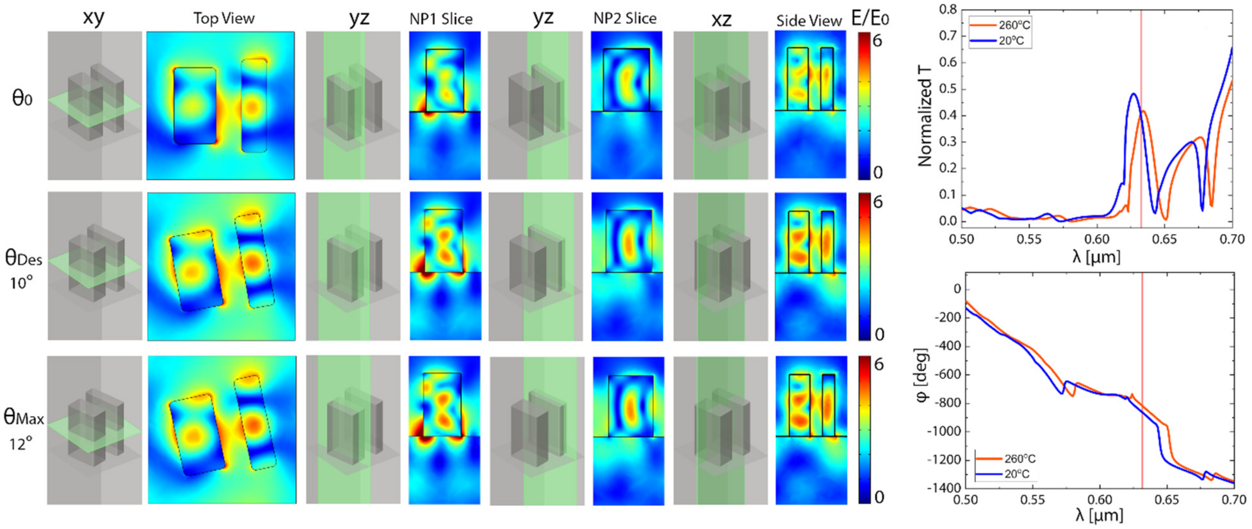

Supplementary Figure 10 – Nanofin #2. Numerical simulation. Electric field profile, transmittance, and phase. a Electric field for top view, side view of left nanopillar, side view of right nanopillar and side view of both pillar from left to right panels respectively. **b** Transmittance and phase spectra for the nanofin #2. at temperatures of 20°C and 260°C. $\boldsymbol{\theta_{Des}}$ is the angle with the highest average phase shift among all the angles used in the design. $\boldsymbol{\theta_{max}}$ is the angle with the highest average phase shift among all the angles.

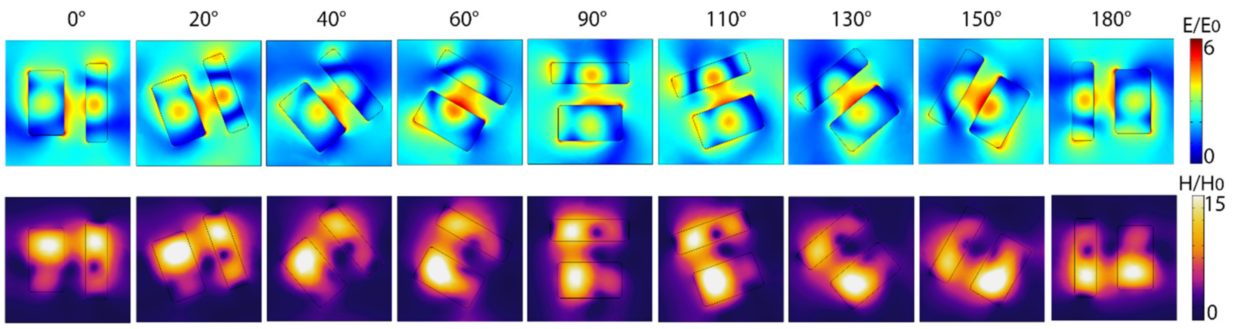

Supplementary Figure 11 – Nanofin #2. 2D electric and magnetic field profile at increasing rotation angle. a Electric filed for top view at increasing angles from 0 deg up to 180 deg for the nanofin #2. **b** Magnetic field from top view at increasing angles from 0 deg up to 180 deg for the nanofin #2.

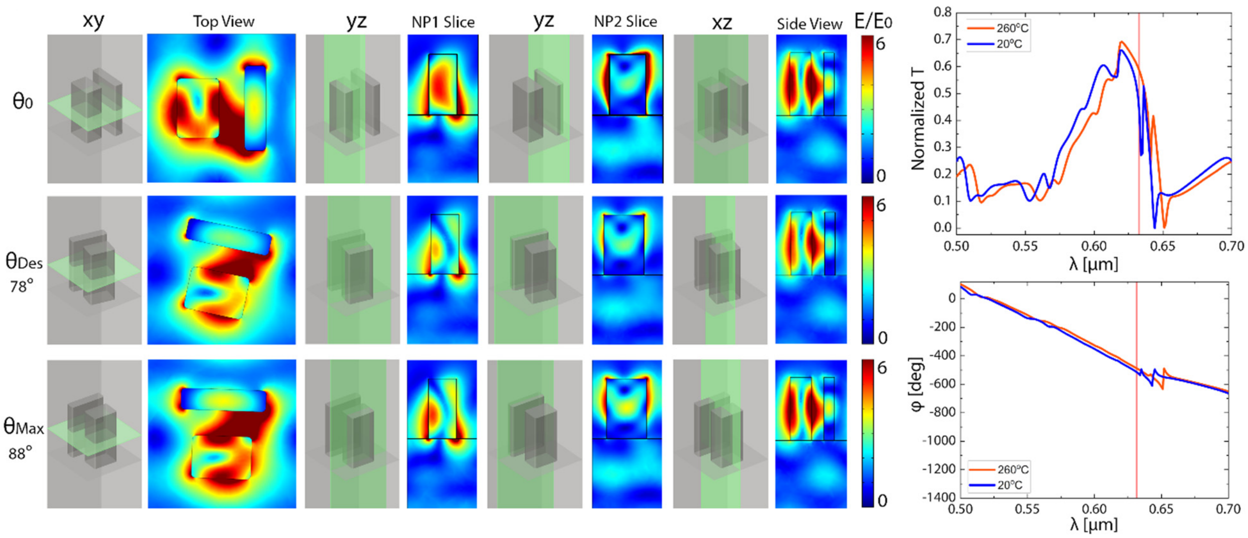

Supplementary Figure 12 – Nanofin #3. Numerical simulation. Electric field profile, transmittance, and phase. **a** Electric field for top view, side view of left nanopillar, side view of right nanopillar and side view of both pillar from left to right panels respectively. **b** Transmittance and phase spectra for the nanofin #3. at temperatures of 20°C and 260°C. $\boldsymbol{\theta_{Des}}$ is the angle with the highest average phase shift among all the angles used in the design. $\boldsymbol{\theta_{max}}$ is the angle with the highest average phase shift among all the angles.

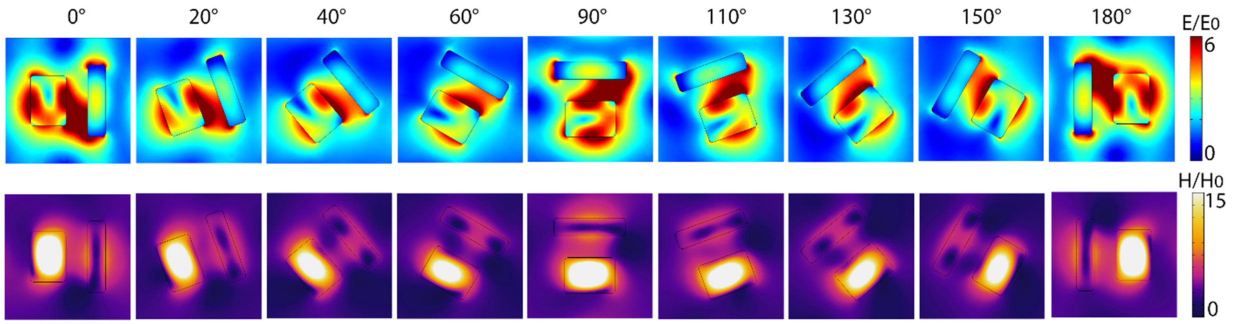

Supplementary Figure 13 – Nanofin #3. 2D electric and magnetic field profile at increasing rotation angle. **a** Electric filed for top view at increasing angles from 0 deg up to 180 deg for the nanofin #3. **b** Magnetic field from top view at increasing angles from 0 deg up to 180 deg for the nanofin #3.

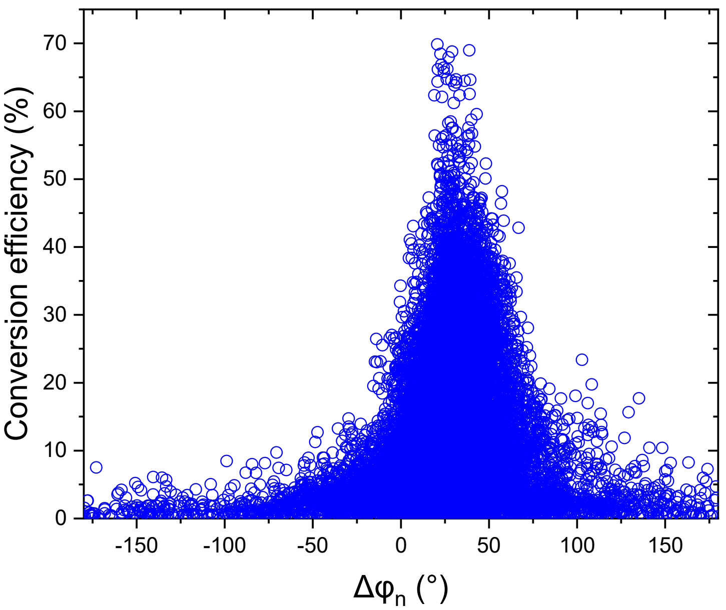

Supplementary Figure 14 – Distribution of conversion efficiency versus phase shift. Here the phase shift is computed as the difference between the phase value at 200°C and the phase value at 20°C: $\Delta\phi_n$(400°C) = ϕ_n(400°C)- ϕ_n(20°C)) of each element. Every circular marker represents a different nanofin geometry.

S5. High NA thermally reconfigurable metalens

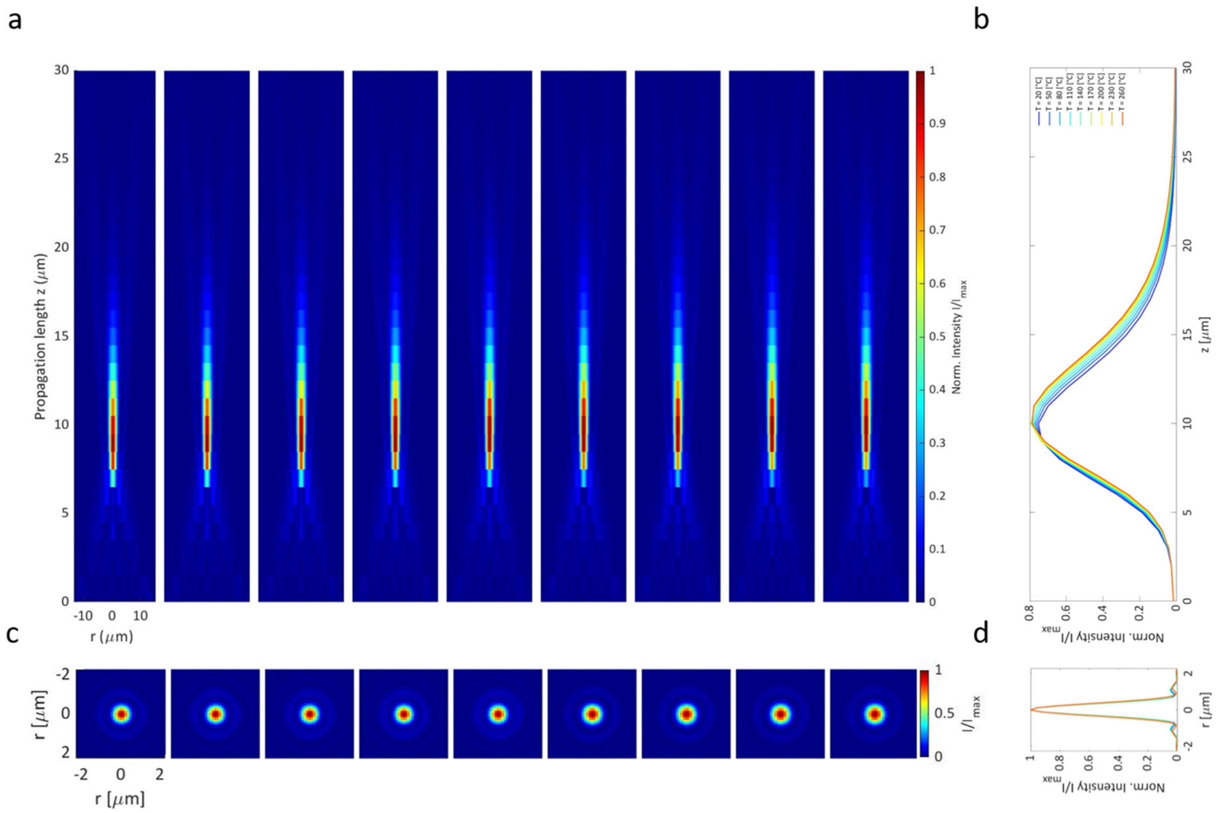

Supplementary Figure 15 – Thermally tunable metalens with NA = 0.4 and focal length variation from $f = 10\ \mu m$ to $f = 11\ \mu m$. **a** Beam propagation focused by the designed ML. 2D intensity profiles along the XZ propagation plane at increasing temperatures. **b** 1D projection of the intensity profiles along the Z propagation direction at increasing temperatures. **c** Point spread function (PSF) 2D profile at the focal plane (XY) at increasing temperatures. **d** 1D projection of the PSF at all simulated temperatures. All the intensity profiles are normalized by the maximum intensity value at each temperature.

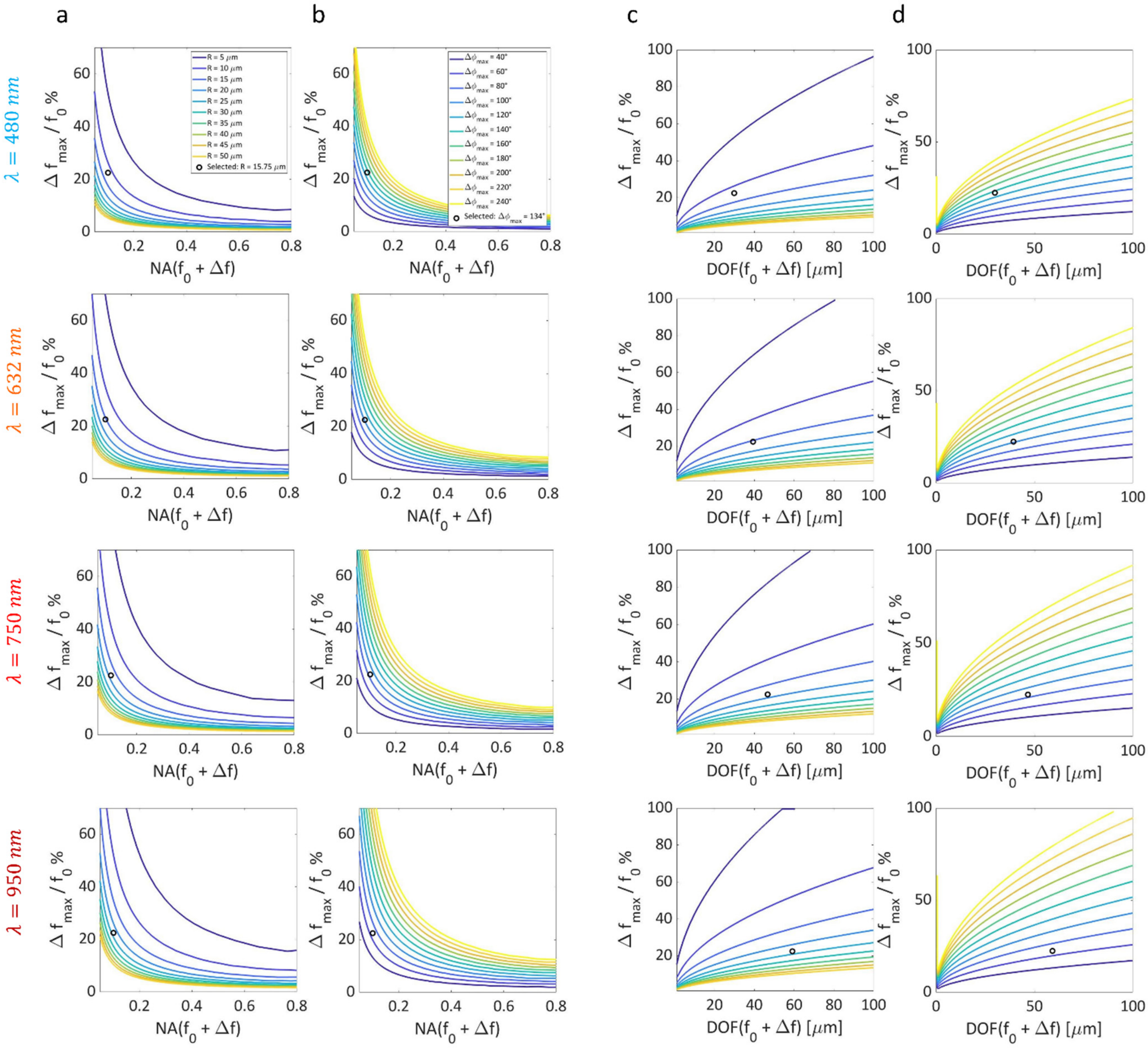

Supplementary Figure 16 – Numerical simulation of the maximum variation of the focal length versus the numerical aperture (NA) and the depth of focus (DOF) at different operating wavelengths. At longer wavelength, higher focal length variations are obtained at higher NA and DOF values.

a Maximum focal length variation over the initial focal length (%) as a function of the numerical aperture NA achievable with a maximum phase variation of $\Delta^{\max}\phi \sim 100$ deg and with different ML radiuses; **b** maximum focal length variation over the initial focal length (%) as a function of NA achievable with a ML radius $R = 15.75\ \mu m$ and with $\Delta^{\max}\phi$ values. **c** Maximum focal length variation over the initial focal length (%) as a function of the DOF achievable with a maximum phase variation of $\Delta^{\max}\phi = 100$ deg and with different ML radiuses; **d** maximum focal length variation as a function of the DOF achievable with a ML radius $R = 15.75\ \mu m$ and with $\Delta^{\max}\phi$ values. The selected value is obtained imposing $f_0 = 200\ \mu m, \Delta f = -45\ \mu m,$ radius $R = 15.75\ \mu m$ and $\Delta^{\max}\phi = max(\phi(r, f_0, \Delta f)) - min(\phi((r, f_0, \Delta f))$, $\Delta^{\max}\phi = 134$ deg at $\lambda = 480\ nm$, $\Delta^{\max}\phi = 102$ deg at $\lambda = 632\ nm$, $\Delta^{\max}\phi = 86$ deg at $\lambda = 750\ nm$ and $\Delta^{\max}\phi = 68$ deg at $\lambda = 950\ nm$.

S6. Thermal-modelling

We have performed COMSOL thermal simulations for a preliminary assessment of the use of a ring micro-heater to achieve a uniform temperature change of the ML. We considered a ML radius of 30µm, a substrate with thermal conductivity of 30 W/mK, representative of sapphire as well as a heater temperature of 260 C. For this preliminary analysis, we consider solely heat conduction, equivalent to a ML placed in an evacuated chamber. Additionally, we do not account for the presence of the silicon meta-atoms because they are discontinuous (no radial heat conduction) and with high thermal conductivity (uniform temperature across the meta-atom). We consider a substrate much larger than the metalens ($r_{sub} = 30*r_{ML}$) and impose a room temperature boundary condition. We observe that for both membrane cases it is possible to achieve a very uniform temperature profile across the lens. To assess the impact of non-uniform temperatures across the metalens, we observe that a 10-degree Celsius difference between the inner and outer nano-scatters would correspond to a variation on the desired phase difference maximum of approximately 3 deg, comparable with the average phase error accumulated during the phase discretization process. Additionally, we observe that even in the non-uniform cases, due to the cylindrical geometry of the studied system, the temperature profile follows a parabolic shape. This can be easily compensated in the design phase by adapting the choice of the meta-atoms to the expected temperature profile. We thus conclude that the use of a micro-ring heater is expected to offer viable experimental implementation of the TR-ML design and operation.

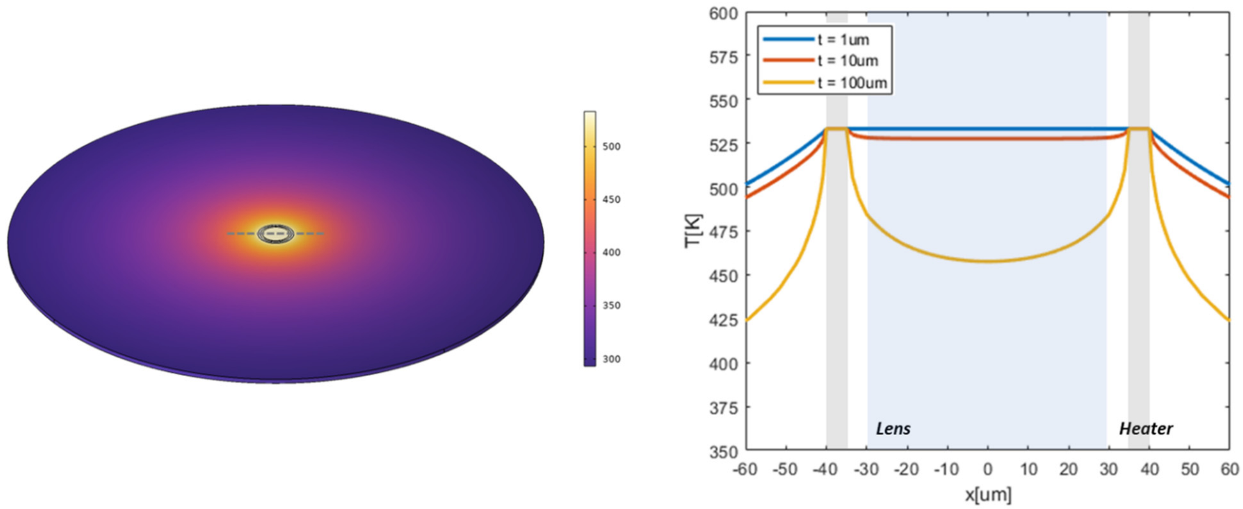

Supplementary Figure 17 – Thermal Modelling. Temperature profile of a 30µm radius metalens at the center of a 10µm thick sapphire membrane surrounded by a micro-ring heater at 260 C. The temperature profiles along the dashed line are shown in the right plot, as a function of the substrate thickness.

References:

1. Chen, W. T., Zhu, A. Y. & Capasso, F. Flat optics with dispersion-engineered metasurfaces. *Nat. Rev. Mater.* **5**, 604–620 (2020).
2. Lalanne, P. Waveguiding in blazed-binary diffractive elements. *J. Opt. Soc. Am. A* **16**, 2517 (1999).
3. Vuye, G. *et al.* Temperature dependence of the dielectric function of silicon using in situ spectroscopic ellipsometry. *Thin Solid Films* **233**, 166–170 (1993).
